# Evaluation of long lasting insecticidal nets in experimental huts and WHO PQT/VCP compliance: A systematic review

**DOI:** 10.1371/journal.pone.0318673

**Published:** 2025-03-12

**Authors:** Divya Teja Koppula, Ananganallur Nagarajan Shriram, Amala Ramasamy, Ashwani Kumar, Manju Rahi

**Affiliations:** 1 ICMR-Vector Control Research Centre, Department of Health Research, Ministry of Health and Family Welfare, Government of India, Medical Complex, Indira Nagar, Puducherry, India,; 2 Centre for Global Health Research, Saveetha (SIMATS) University, Chennai, Tamil Nadu, India; World Health Organization, Regional Office for South-East Asia, INDIA

## Abstract

Malaria control in highly endemic regions relies heavily on vector control tools, particularly LLINs. The effectiveness of LLINs varies by eco-epidemiological conditions and brands. A comprehensive review of WHO interim-approved LLIN brands is necessary to address this variability. This systematic review screened 145 articles, refining them to 27 eligible publications, to assess the efficacy of WHO-recommended LLINs, focusing on synthetic pyrethroids and synergists like Piperonyl Butoxide or Pyriproxyfen. The review demonstrated that LLINs impregnated with synthetic pyrethroids, especially when used with synergists, are more effective than regular LLINs. However, adherence to WHO PQT/VCP (World Health Organization Prequalification Unit/Vector Control Product) checklists was inconsistent. Several LLIN pairs which includes candidate and comparator nets showed equal efficacy (100% in both arms), including Olyset Plus vs. Olyset Net, DuraNet vs. PermaNet 3.0, Interceptor G2 vs. Interceptor, MagNet vs. DuraNet, Dawa Plus 3.0 vs. Dawa Plus 2.0, and Veerralin vs. PermaNet 3.0 in terms of mosquito mortality. Significant efficacy differences were noted between various bed net pairs: Interceptor vs CTN (RR: 1.5, 95% CI: 1.28-1.66); Olyset Net Duo vs Olyset Net (RR: 1.1, 95% CI: 1.01-1.18); ICON Maxx vs CTN polyester (RR: 7.7, 95% CI: 3.6-16.31); Dawa Plus 3.0 vs Dawa Plus 2.0 (RR: 2.1, 95% CI: 1.34-3.15); Interceptor G2 vs Interceptor G1 (RR: 0.9, 95% CI: 0.77-0.96). These findings inform the development of improved net designs and ensure alignment with WHO guidelines, enhancing vector control measures. The review supports improved malaria control strategies and sustained LLIN utilization, highlighting the need for LLIN manufacturers to align with WHO guidelines.

## Introduction

Malaria, an age-old affliction with a long history of tormenting humanity, remains a formidable global health concern, especially in the tropical and subtropical regions. Historical accounts from ancient civilizations document their impact on human populations, emphasizing their enduring presence and harmful effects. The correlation between swampy environments, ill health, and enlarged spleens, as recorded in Hindu and Greek literature from as early as the 6th century BC, highlights the long-standing acknowledgment of the impact of malaria [1]. In tropical and subtropical regions around the world, malaria is the most prominent vector-borne disease, rapidly spreading through populations [2].

Ronald Ross achieved a significant breakthrough in malaria research in 1897. He discovered a malarial pigment in an *Anopheles* mosquito that had ingested blood from an infected individual, indicating that the mosquito functions as a carrier of malaria parasites. This critical period marked a significant advancement in our understanding of malaria transmission [3]. Approximately 537 recognized *Anopheles* species are present; nonetheless, only 30-40 of them can transmit malaria, that is, acting as vectors [4]. Vectors, which are frequently hematophagous insects, transmit diseases by ingesting pathogens while feeding on the blood of an affected host and subsequently transmitting the parasite to other victims. Once infected, vectors can continue to transmit the disease with each subsequent bite throughout their lifespan [5].

The female mosquito of the *Anopheles* genus transmits the parasite; the protozoan parasite responsible for causing malaria belongs to the genus *Plasmodium* [6]. Four species commonly recognized as human parasites within the Plasmodium genus include *Plasmodium malariae*, *P. ovale*, *P. vivax*, *P. falciparum,* and *P.knowlesi* [7,8]. The disease transmission by *An*. vectors remains stable despite numerous established explanations of how parasites reproduce within *Anopheles* mosquitoes.

The World Malaria Report documented 263 million reported cases of malaria worldwide in, with an estimated 597,000 malaria-related deaths in 2023, slightly lower than the 252 million cases reported in 2022 [9]. In 2022 India accounts for 66% of global burden and 46% of these cases were *P.vivax* [10].

In India, efforts to eradicate this vector-borne disease (VBD) began in the 1900s, primarily through anti-larval operations. Pyrethrum sprays were introduced in the 1930s, followed by the documented effectiveness of Dichloro-diphenyl-trichloroethane (DDT) in the 1940s. In 1953, the government launched the National Malaria Control Programme (NMCP), which included prominent activities such as Indoor Residual Spraying (IRS), case monitoring and surveillance, and the provision of antimalarial treatment for patients. [11,12]. The elimination of malaria targets anopheline species using specific vector control tools include insecticide-treated nets (ITNs), long-lasting insecticidal nets (LLINs), IRS [13].

In 2016, the Government of India launched the National Framework for Malarial Elimination (NFME) in collaboration with the National Center for Vector Borne Disease Control (NCVBDC). The vision of NFME is to “eliminate malaria nationally and contribute to improved health, quality of life, and poverty alleviation.” Also to achieve a sustained absence of indigenous malaria cases and deaths for three years, paving the way for WHO certification of malaria elimination [14].

To date, 42 countries have achieved WHO certification for being malaria free. Recent additions to the list include El Salvador, Algeria, Argentina, Paraguay, and Uzbekistan [15]. In 2023, Belize, followed by Azerbaijan and Tajikistan, achieved malaria-free status, In 2024, Cabo Verde and Egypt received this recognition [16].

According to the Center for Disease Control and Prevention (CDC), malaria interventions have saved millions of lives and reduced the mortality rate by 36% between 2010 and 2020, instilling hope for the elimination of malaria [17].

### History of vector control tools

The use of mosquito net dates back to ancient times as a means of protection against various insects. During World War II, soldiers utilized treated bed nets with residual insecticides to combat VBDs like malaria and leishmaniasis [18].

As part of the RBM (Roll Back Malaria) initiative, ITNs were implemented in the 1980s. These nets successfully lowered malaria incidence, morbidity and overall infant death rate due to malaria [19]. In the early 1990s, to definitively determine the role of bed nets in malaria prevention, Dr. Godal, affiliated with the Special Programme for Research and Training in Tropical Diseases (TDR), made a path breaking decision by allocating the entire TDR malaria research budget to large-scale trials. to comprehensively evaluate the efficacy of ITNs in reducing malaria mortality [20]. However, ITNs had several limitations. They were not effective against exophagic malaria vectors, and their coverage remained low, specifically among children and pregnant women who were at high risk. Additionally, they required frequent re-treatment with insecticides, a costly and challenging task, particularly in rural areas [21,22]. With the invention of LLINs, a solution to these challenges emerged. Even after 20 washes, these nets maintain their insecticidal action for up to three years [23].

### Methods of insecticide loading

Two main approaches are implemented for loading insecticides in nets:

a) **Incorporation technology:** This method involves integrating a pyrethroid insecticide into a polyethylene netting material, where it migrates to the fiber surface through specific reagents. If the surface insecticide washes off, this migration process allows the insecticide to regenerate from a reservoir.
b) **Coating technology:** This method utilizes a polyester multifilament netting material with a polymer coating containing the insecticide, acting as a reservoir to replenish the surface insecticide [23].

The United Nations International Children’s Emergency Fund (UNICEF) has declared LLINs as one of the most effective tools for malaria prevention, functioning as both physical and chemical barriers against malaria vectors. Sub-Saharan Africa is responsible for more than 90% of the world’s malaria cases, however, as a result of LLINs, the incidence has declined by 50% in that region. Sumika Life-Tech Co., Osaka, Japan, developed Olyset Net®, the first LLIN using incorporation technology, which is pre-treated with permethrin and known to remain effective for at least three years [24,25]. Table 1 represents WHO approved LLINs with concentrations and their manufacturers.

**Table 1:** WHO approved LLINs with concentrations and their manufacturers.

S. No	Year	Name of the Brand	Concentrations	Manufacturers	Country
1.	2001	Olyset^TM^ Net	permethrin with 2% w/w	Sumitomo Chemical Co., Ltd.	Japan [26]
2.	2004	PermaNet®	deltamethrin with 55 mg a.i./m^2^	Vestergaard-Frandsen	Denmark [27]
3.	2006	Interceptor®	alpha-cypermethrin with 200 mg a.i./m^2^.	BASF	Germany [28]
4.	2007	Netprotect®	deltamethrin with 63 mg a.i./m^2^	Intelligent Insect Control	France [29]
5.	2007	DuraNet©	alpha-cypermethrin with 221 mg/m^2^ or target dose of 5.8 g/kg AI.	Clarke Mosquito Control	USA [29]
6.	2007	DawaPlus®	deltamethrin with 40 mg a.i./m^2^	Tana Netting in collaboration with Bayer Environmental Science	Thailand [29]
7.	2008	Permanet® 2.0 (poly-filament polyester fiber)	deltamethrin with 55 mg a.i./m^2^	Vestergaard-Frandsen	Switzerland [30]
8.	2008	Permanet® 3.0	deltamethrin with 4.0 g a.i./kg; Side panels 2.8 g a.i./kg and PBO with 25 g a.i./kg	Vestergaard-Frandsen	Switzerland [30]
9.	2008	Permanet® 2.5	deltamethrin with 115 mg a.i./m^2^ for boarders and 85 mg a.i./m^2^ for side panels and roof.	Vestergaard-Frandsen	Switzerland [30]
10.	2008	ICON® MAXX	lambda-cyhalothrin with 50mg a.i./m^2^	Syngenta	Switzerland [30]
11.	2009	Olyset® 2.0 (high-density monofilament polyethylene)	permethrin with 2% w/w	Sumitomo Chemical Co., Ltd.	Japan [31]
12.	2009	DawaPlus®2.0	deltamethrin with 80 mg a.i./m^2^	Tana Netting	Thailand [31]
13.	2009	Yorkool®	deltamethrin with 56 mg a.i./m^2^	Tianjin Yorkool International Trading Co., Ltd	China [31]
14.	2011	LifeNet®	deltamethrin with 340 mg a.i./m^2^	Bayer Crop Science	France [32]
15.	2011	MAGNet ^TM^ LN	alpha-cypermethrin with ± 25% of 5.8 g AI/kg	V.K.A Polymers	India [32]
16.	2011	Royal Sentry® LN	alpha-cypermethrin with 5.8 g AI/kg ± 25%	Disease Control Technologies	USA [32]
17.	2011	Yahe® LN	deltamethrin with ± 25% 1.35 g AI/kg-2.25 g AI/kg	Fujian Yamei Industry	China [32]
18.	2012	OlysetPlus	permethrin with 2% w/w and PBO with 1% w/w	Sumitomo Chemical Co., Ltd.	Japan [33]
19.	2013	Netprotect	deltamethrin with 68.4 mg/m^2^	Bestnet A/S	Denmark [34]
20.	2013	DuraNet	alpha-cypermethrin with 221 mg/m^2^	Shobikaa Impex	India [34]
21.	2015	MiraNet	alpha-cypermethrin with 180 mg/m^2^	A to Z Textile Mills Ltd.	United Republic of Tanzania [35]
22.	2015	PandaNet 2.0	deltamethrin with 76 a.i./m^2^	Life Ideas Textiles Company Ltd.	China [35]
23.	2015	Yahe LN	deltamethrin with 55.5mg a.i./m^2^	Fujian Yamei Industry	China [35]
24.	2015	SafeNet LN	alpha-cypermethrin with 6.7 g AI/kg or 5.0 g AI/kg	Mainpol GmbH	Germany [35]
25.	2016	Veeralin	alpha-cypermethrin with a.i./m^2^ and PBO with 79.2 mg/m^2^	Vector Control Innovations Pvt Ltd	India.[36]
26.	2017	Interceptor G2 LN	alpha-cypermethrin with 100 mg/m2 and chlorfenapyr 200 mg/m^2^	BASF	Germany [37]
27.	2017	DawaPlus 3.0 LN	deltamethrin with 3 g a.i./kg and PBO with 11 g/kg; side panels with 2.5 g a.i./kg	Tana Netting	United Arab Emirates [37]
28.	2017	DawaPlus 4.0 LN	deltamethrin with 3 g a.i./kg and PBO with 11 g/kg;	Tana Netting	The United Arab Emirates [37]

#### Assessment of *LLIN’s* efficacy in experimental hut trials (EHT).

To assess the performance of *LLIN’s* under controlled conditions against pyrethroid-susceptible malaria vectors, small-scale studies are necessary, such as the EHT recommended by the *WHO* is one of the criteria used to assess LLIN’s efficacy The following are the outcome measures of the EHT.

(i) ***Deterrence* (D):** This refers to the percentage decrease in the entry of malaria vectors into the hut compared to control huts (untreated nets).
(ii) ***Exophily* (E):** This measures the percentage of malaria vectors caught in the exit and veranda traps.
(iii) ***Blood-feeding Inhibition* (BFI):** This indicates the percentage reduction in blood-feeding compared to control nets.
(iv) ***Mortality* (Mo) (immediate or delayed):** The percentage of malaria vectors that perished upon entering the hut, either by morning or after being caught alive and held for 24 h with a sugar solution.

*D* and *BFI* act as personal protection measures. *BFI* and *M* of the candidate net should be equal to or greater than those of reference *LLINs*. The type and content of insecticides have an impact on insecticidal activity. The insecticidal content was measured either in *g/kg* or *mg/m*^*2*^, according to *WHO* criteria. This information helps interpret the bioefficacy data [38,39]. Mosquito Mortality (MM) is another criterion used to assess *LLINs* efficacy through cone bioassays, if needed, in tunnel tests.

While numerous brands of *LLIN*s have been assessed *via* Phase II *EHT*, no systematic reviews have attempted to evaluate their efficacy at this stage. According to the WHO PQT/VCP II guidelines, this manuscript comprehensively examines the effectiveness of *LLIN*s in experimental hut evaluations. While numerous LLIN brands have been tested via Phase II EHTs, systematic reviews evaluating their efficacy are lacking. The aim of this review was to assess the efficacy of *LLIN*s impregnated with synthetic pyrethroids and synergists, such as PBO or PPF, in controlling malaria across various eco-epidemiological regions in terms of outcome measures of EHT and mosquito mortality.

### Significance of this review

This information is vital for policymakers to implement effective vector control management, especially concerning the synthetic pyrethroid net’s efficacy against malaria vectors after 20 washes. This research endeavor seeks to bridge existing knowledge gaps regarding *LLIN*s efficacy and its usage.

## Materials and methods

**Fig 1:**
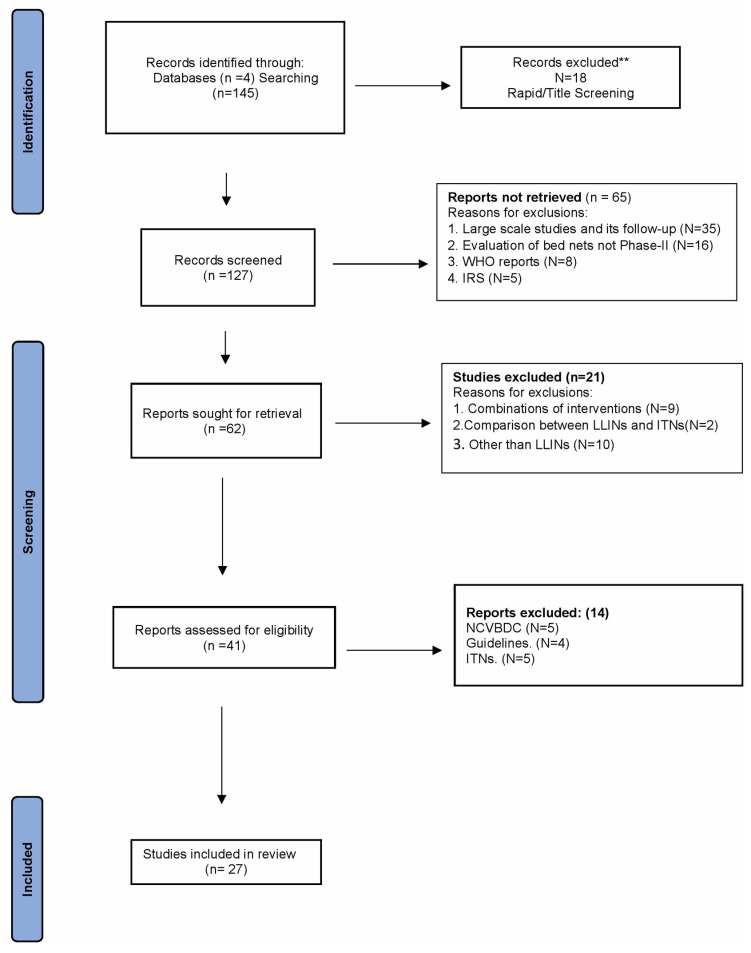
PRISMA Flowchart.

In this review, we concentrated on the scientific literature, encompassing all articles pertaining to Phase II experimental hut evaluations. The PRISMA 2020 Checklist is shown in S3 File. This Systematic Review was not registered however, we adhered to the Preferred Reporting Items for Systematic Reviews and Meta-Analyses (PRISMA) criteria to ensure a transparent review procedure. Fig 1 depicts the study selection process using a PRISMA flow chart.

### Search methods for identification of studies

The literature search for this review was conducted between September 2022 and March 2024 across various databases, including PubMed, Google Scholar, and Web of Science, using a Boolean search strategy (AND, OR, NOT) with the following search terms.

1. ((Phase II experimental Hut trial*) AND (*LLIN*s OR “Long-lasting insecticidal nets”)).
2. (“Long-lasting Insecticide net*”) OR (“Long lasting Insecticidal Net*”)
3. (Phase II experimental Hut trial*)

The search yielded 145 articles from all databases (Shee1 in S1 File). These studies evaluated the protective efficacy of various types of *LLIN*s containing specific insecticides against human volunteers, making them suitable for Phase 2 trials. The narrowing process involved careful consideration, resulting in the selection of 27 articles that met the inclusion criteria for further data extraction and analysis. We synthesized the results to provide a comprehensive overview of the evidence regarding the protective efficacy of *LLIN*s in selected experimental hut studies.

### Inclusion criteria

Criteria for including articles were as follows:

1. Articles focused solely on Phase II trials explicitly conducted through WHO PQT/VCP II
2. Articles discussing the efficacy of various types of *LLIN*s, their fabric types, and their compositions.
3. Information is provided about the insecticides utilized for *LLIN* application and their concentrations.
4. Studies that were published in English.

### Exclusion criteria

Excluded articles fell under the following categories:

1. Large- scale studies evaluating *LLIN*s but not specifically Phase-II trials.
2. Guidelines and protocols, such as those from the WHO, and NCVBDC.
3. Articles discussing interventions other than *LLIN*s, such as ITNs, IRS, Insecticide-Treated Wall Linings (ITWL), Long-Lasting Insecticidal Blankets (LLIB).
4. Studies involving combinations or comparisons between interventions, such as *LLIN*s with IRS, or Long-Lasting Wall Linings (LLWL) or organophosphates.

These criteria ensured the selection of only articles that focused on Phase II trials of *LLIN*s.

#### WHO PQT/VCP checklist.

We screened each selected study for adherence to the WHO PQT/VCP guidelines using a brief checklist that included the following key criteria:

- Inclusion of study arms with candidate net, a reference net known as a positive control net, and an untreated net known as a negative control.
- Use of experimental huts that meet the structural specifications outlined by the WHO PQT/VCP.
- Involvement of adult human volunteers during the experimentation.
- Employment of either the cone test or the tunnel test to assess net efficacy.
- Reporting on the wash resistance of the candidate net.
- Detailed explanation of the primary outcome measures used in EHTs, such as deterrence, exophily, blood-feeding inhibition, and mortality.

## Statistical analysis

The primary objective of this review was to analyse the efficacy of *LLIN*s by assessing the percentage *MM* and outcome measures in Phase II experimental hut trials. For each selected study, we reported the Relative Risk (RR) along with its 95% confidence interval (CI), representing the ratio of *MM* risk in the candidate net compared to the control net. Furthermore, we included a forest plot to visually depict the comparison of various bed nets with the comparator nets.

Additionally, we assessed the risk of bias (ROB) to evaluate the reliability of individual studies included in the analysis, as well as for visual presentation of studies adhering to the WHO PQT/VCP checklist using robivs The studies were classified as’ low risk,’ some concerns,’ or ‘high risk’ of bias based on the aforementioned checklist. This allowed us to thoroughly evaluate the quality of evidence presented in the review process.

### Data extraction

The data were extracted by two independent reviewers. The articles were screened for full-text review to determine their eligibility for inclusion in the analysis. We developed a standardized data extraction form to facilitate a comprehensive understanding of the long-lasting insecticidal net (*LLIN*s) research landscape. This form captured key study details, including the authors’, names, year of publication, candidate and comparator nets, testing methods, mosquito species examined, mortality rates observed in the experiments, and *EHT* outcome measures. To ensure the effectiveness of *LLINs*, the fabric must be durable, comfortable, and resistant to use, while the insecticide should be applied at an optimal concentration for sustained control of malaria vectors. The findings for the respective LLINs are presented in S1 and S2 Tables.

## Results of systematic review

After applying the search strategy, out of 145 studies, 18 were excluded during Rapid/Title Screening. Subsequently, upon screening titles and abstracts, 127 articles were found relevant to the objective. Following these, 65 studies were excluded due to their focus on large-scale evaluations of *LLIN*s rather than Phase II trials, using IRS as intervention and protocols. Of the remaining 62 studies, 21 were further excluded for involving combinations of interventions such as *LLIN*s with IRS, ITNs, ITWL, or with other interventions like LLIB, Insecticide Treated Blanket (ITB), Conventionally Treated Net (CTN), Impregnated Bed Nets (IBN), Insecticide Treated Wall Hangings (ITWH) and other interventions rather than *LLIN*s. From the remaining 41 studies, 14 were eliminated for the following guidelines, other than the WHO PQT/VCP. Thus, studies meeting predefined eligibility criteria were used for the final review of a practice to enhance the reliability and validity of the review (Sheet 1 in S1 File, and S2 File).

Fig 1 illustrates the search strategies employed in selecting the studies. The Preferred Reporting Items for Systematic Reviews and Meta-Analyses (PRISMA) guidelines ensure the transparency and consistency in reporting systematic review of LLINs thereby enhancing its overall quality and reliability. PRISMA chart describes the process undertook in this review. This approach ensures a systematic and transparent process in selecting, evaluating, and reporting the findings of a systematic review

### Description of included studies

Among the entire spectrum of 27 articles chosen, their collective scope encompassed the performance analysis of 24 distinct *LLIN*s across the global landscape. Noteworthy *LLIN*s featured in this corpus include experimented on *LLIN*s. The most prominently studied LLIN brands and their respective number of studies: PermaNet 2.0 (*k* = 6), PermaNet 3.0 (*k* =  6), PermaNet Dual (*k* = 1), Olyset Net (*k* = 6), Olyset Plus (*k* = 4), Olyset Duo (*k* = 2), ICON Max (*k* = 1), Netprotect (*k* = 1), DuraNet (*k* = 7), Interceptor (*k* =  4) and Interceptor G2 (*k* =  2), Yorkool (*k* = 1), LifeNet (*k* = 2), Dawa Plus 2.0 (*k* = 2), Dawa Plus 3.0 (*k* = 2), Dawa Plus 4.0 (*k* = 2), MAGNet (*k* = 4), MiraNet (*k* = 1), Veeralin (*k* = 1), SafeNet (*k* = 1), SafeNet NF *(k* = 1), Yahe (*k* = 1), PandaNet 2.0 (*k* = 1), and Royal Guard (*k* = 1). Killing effects (min, max): PermaNet 3.0 (10.4, 90), PermaNet Dual (104,104), Olyset Net (8.7, 96.3), Olyset Plus (36.9, 97.2), Interceptor (11, 75.9), and Interceptor G2 (41, 82), LifeNet (70, 91.3).

### Findings

PermaNet 3.0, showed enhanced efficacy against resistant mosquito vector populations, attributed to a higher concentration of deltamethrin compared to PermaNet 2.0, as observed in Tanzania, and with Olyset Plus and Yorkool in Kolokope Togo [40,41]. In addition, PermaNet ® Dual was 88% more effective than PermaNet 3.0 and 2.0, which is why it was made of polyester and treated with pyrrole chlorfenapyr and pyrethroid deltamethrin [42].

Following bioassay and experimental hut assessments, Malima et al. (2013) established that the interceptor aligns with WHO approval criteria and is recommended for use against malaria vectors [43]. Upon comparative analysis with four other *LLIN*s, Interceptor demonstrated a higher efficacy rate [44]. Notably, Interceptor G2, which has two active ingredients, killed more pyrethroid-resistant malaria vectors than the other mosquito nets. This was a big step forward in field evidence gathering in Cote d Ivoire and Tanzania [45,46].

Combining Olyset Net Duo and PPF results in significantly improved performance and quality in malaria transmission control [47,48]. Combining it with PBO also resulted in a similar enhancement, which led to Olyset Plus delivering improved results during Phase II evaluations [49,50,41,51]. Ketoh et al. (2018) and Toe et al. (2018) found that Olyset Plus was effective in killing pyrethroid-resistant malaria vectors [41,51]. Pennetier et al. (2013) corroborated Olyset Plus’s efficacy, while acknowledging the need for further evaluation [49].

Deltamethrin-treated Netprotect and PermaNet 2.0 were less effective than alpha-cypermethrin-treated DuraNet and Interceptor. [44]. Mahande et al. (2018) demonstrated DuraNet’s efficacy in controlling wild mosquito populations [52]. However, an Ethiopian study presented a contrary perspective, revealing DuraNet’s lower-to-moderate efficacy against pyrethroid-resistant populations [53]. Ketoh et al. (2018) revealed that yorkool has a notably lower efficacy rate [41].

In Phase II evaluations, the LifeNet polypropylene net with deltamethrin alignment met the WHO efficacy criteria for effectiveness. It was shown to be effective against *Anopheles fluviatilis* susceptible populations in India and against *An. gambiae* resistant population in Benin [54,55]. Although Dawa Plus 2.0, exhibited reduced efficacy [51], Bayili et al. (2019) ‘s experiment incorporating PBO transformed it into Dawa Plus 3.0, and Dawa Plus 4. 0, which met the WHO Health Organization criteria. Notably, Dawa Plus 4.0 exhibited significantly enhanced protection against vector populations in the studied area [56]. In contrast, phase II evaluations conducted in India showed that neither Dawa plus 3.0 nor Dawa plus 4.0 met the WHO criteria [57]. Tungu *et al.* (2015) performed phase II evaluations on the ICON Max Net and confirmed that this net provides significant protection in terms of mortality [58].

Phase II evaluations spanning India, Tanzania, and Cote d Ivoire underscored MAGNet’s exceptional performance and WHO compliance [59,60,61]. Even when confronted with high resistance levels, MiraNet showed commendable protection against mosquito vectors [61]. Phase II evaluations conducted in India and Tanzania have reported Veeralin *LLIN*s’ exceptional performance of both Yahe® LN and Panda® Net 2.0 *LLIN*s worked well against pyrethroid-resistant malaria vectors, which meets the WHO requirements for moving on to phase III trials in communities [62,63,64]. The Royal Guard met the WHO criteria for exhibiting efficacy against *Anopheles gambiae*.[65] According to Azizi et al. (2021), SafeNet NF® and SafeNet® *LLIN*s offer protection on par with the interceptor [66] (Sheet 2 in S1 File).

**Fig 2:**
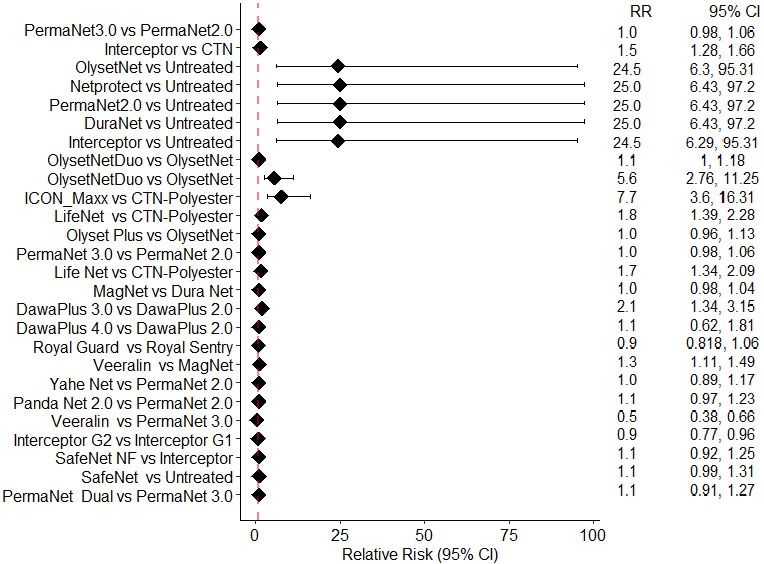
Relative risk forest plot.

Fig 2 represents the Relative Risk (RR) of mosquito mortality for each candidate net in comparison to its counterpart net, aiming to gauge the efficacy of the candidate bed nets. In this context, the Relative Risk is computed as the ratio of the risk of mosquito mortality in the candidate net to that of the comparator net. An RR of 1 signifies no disparity in Mosquito Mortality (MM) risk between the two brands. An RR > 1 implies that the candidate net is linked to a heightened risk of mosquito death compared with its counterpart. Fig 2 does not entirely represent the studies included in this review. We specifically excluded studies that met two conditions: (i) they showed no difference in the risk of MM between the candidate and comparator nets, and (ii) they reported no MM (untreated nets) [41,61].

Based on the observed RR values, several pairs of bed nets were found to be equally effective (RR = 1, indicating 100% efficacy in both arms): Olyset Plus vs. Olyset Net [50], DuraNet vs.PermaNet 3.0 [52], Interceptor G2 vs. Interceptor [45], MagNet vs. DuraNet [59], and Dawa Plus 3.0 vs. Dawa Plus 2.0, Dawa Plus 4.0 vs. Dawa Plus 2.0 [56] (Sheet 3 in S1 File).

Additionally, significant differences in efficacy were observed between the following pairs of bed nets: Interceptor vs CTN, RR: 1.5 (95% CI: 1.28, 1.66) [43]; Olyset Net Duo vs Olyset Net, RR: 1.1 (95% CI: 1.01, 1.18),RR: 5.6 (95% CI:2.76,11.25) [47,48]; ICON Maxx vs CTN polyester, RR: 7.7 (95% CI: 3.6,16.31) [58]; Dawa Plus 3.0 vs Dawa Plus 2.0, RR: 2.1 (95% CI: 1.34, 3.15) [57]; and Interceptor G2 vs Interceptor G1, RR: 0.9 (95% CI: 0.77, 0.96) [46].

Furthermore, it is noteworthy that although the same brand of bed nets was compared in two different locations, Benin and Côte d’Ivoire [47,48], the efficacy varied significantly between the two places, with RR values of 1.01 (95% CI: 1.01, 1.19) and 5.6 (95% CI: 2.76, 11.2), respectively. However, to establish this heterogeneity with respect to location, additional studies comparing the same brands of bed nets are required.

Fig 2 shows that different brands of *LLIN*s are highly heterogeneous in terms of efficacy. One of the reasons could be that reference nets were different in each study. This shows how important it is to carefully choose *LLIN*s for controlling malaria vectors.

#### Risk of bias.

Our systematic review revealed that adherence to the WHO PQT/VCP checklists was not always consistent. We conducted potential bias assessment by two independent reviewers and in case of any disagreements, which were dissolved by discussion. Out of the 27 studies included in the assessment of risk of bias, we assessed 9 studies were unclear in terms of non-adherence to few a parameter, and 18 studies were low risk of bias (Sheet 4 in S1 File). Here, we discuss key areas of concern:

#### Study arms.

While the inclusion of negative and positive controls is commendable for comparison, inadequate definition of the positive control’s specifications (insecticide, treatment technique, netting material, denier, mesh size) can introduce bias and skew comparisons.

#### Experimental huts.

Utilizing experimental huts is a strength, mimicking real-life conditions for *LLIN* evaluation. However, inconsistencies in hut structural features across studies can bias mosquito collection.

#### Human volunteers.

The use of human volunteers enhances realism, but potential bias arises from non-representative volunteer selection or uncontrolled factors like individual attractiveness to mosquitoes.

#### Testing methods.

Standardized WHO cone bioassays and tunnel tests provide a consistent approach. Variations in testing conditions or execution, however, can introduce bias.

#### Wash resistance criteria.

Defined criteria for assessing net efficacy after 20 washes are a positive step, but a lack of data on actual performance leaves the risk of bias unclear (S3 Table).

Implementing a color-coded system (yellow: unclear, green: low, red: high) to highlight areas of concern in the conduct of trial can effectively communicate potential bias levels.

This comprehensive review ensures the consideration of potential bias in the studies, resulting in more precise conclusions about the effectiveness of *LLIN*s in controlling malaria vectors. A traffic light plot for visualizing the risk of bias across these key elements was generated using Robvis, an R Shiny Dashboard [67].

**Fig 3:**
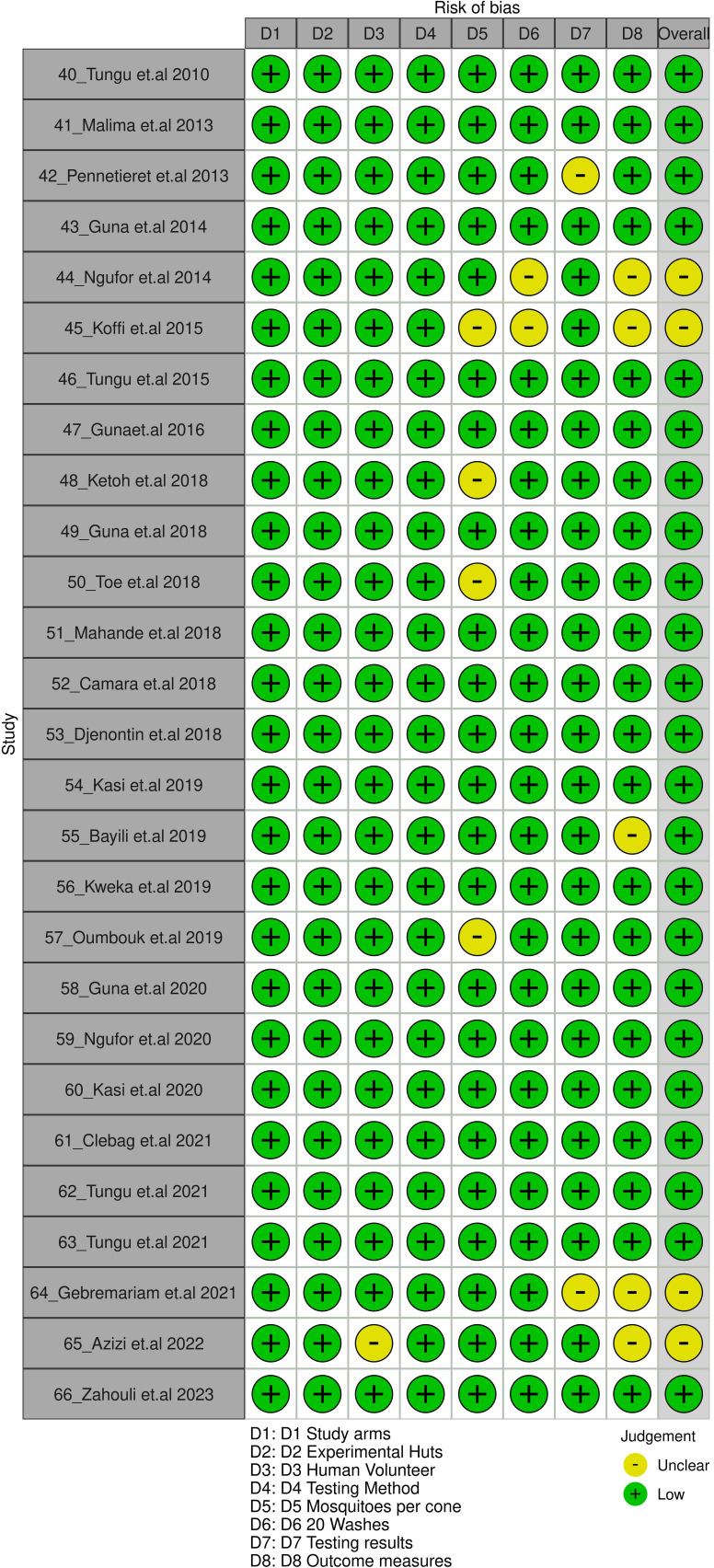
Traffic light plot.

This standardized approach ensured a consistent evaluation of *LLIN* efficacy across diverse studies. Consequently, we could perform meaningful comparisons and draw robust conclusions regarding their effectiveness in malaria vector control. Furthermore, tailoring the bias assessment to each study design’s specific characteristics and potential biases enhanced the overall analysis’s validity and robustness. Fig 3 shows traffic light plot for adherence to WHO PQT/VCP.

## Discussion

This systematic review examines how well *LLIN*s work in small-scale Phase II trials. It uses existing research to lay the groundwork for creating new mosquito control strategies that people want to use for a long time.

This review investigates the efficacy of various types of *LLIN*s, focusing on those treated with synthetic pyrethroids. It explored their efficacy in killing malaria vectors, repelling them (deterrence), preventing them from feeding on people (blood-feeding inhibition), and mosquito behaviour of staying outdoors (induced exophily). This review considered studies conducted in diverse ecological settings across West Africa, Tanzania, India, and Ethiopia with a focus on *LLIN*s recommended by the WHO.

PermaNet 2.0 and PermaNet 3.0, are mosquito nets with different concentrations of insecticide [40,41]. Both nets killed more malaria vectors that were not resistant to pyrethroids (pyrethroid-susceptible). PermaNet 3.0 exhibits higher mosquito mortality rates after 20 washes against *An. gambiae* owing to its elevated insecticide concentration and the inclusion of an additional ingredient, namely PBO [40,41], a synergist that enhances the efficacy of insecticides, such as deltamethrin, and suppresses the enzyme defense mechanisms of vector mosquitoes [68]. However, in resistant insects with elevated enzyme levels, PBO’s impact of PBO may be less pronounced initially, but can still increase sensitivity to insecticides over time by inhibiting these enhanced metabolic systems. [69]

Studies comparing different mosquito nets revealed that the Olyset performed comparably well with other nets (Netprotect, PermaNet 2.0, DuraNet, and Interceptor). While all of these nets effectively deterred malaria vectors and inhibited feeding, they differed in terms of inducing vector mosquitoes to remain outdoors (exophily) and mortality rates, indicating they were not effective against exophagic malaria vectors. Olyset nets, treated with permethrin, induced higher levels of exophily, causing malaria vectors to remain outside more frequently compared to other nets. In contrast, nets treated with alpha-cypermethrin demonstrated higher mosquito mortality rates. Alphacypermethrin-treated LNs are more effective in killing malaria vectors, thereby reducing disease transmission risks and the breeding population. Unlike permethrin-treated LNs, which induced exophily, alpha-cypermethrin strikes a balance by effectively killing of malaria vectors while minimizing dispersion. This dual action ensures a more substantial and sustained impact on vector control. [44].

The regeneration capacity of Olyset Plus was better than that of the regular Olyset nets after washing. Studies in Benin revealed higher overall mortality rates with Olyset Plus, highlighting the need for larger studies to determine its duration and user acceptability [49].

Trials conducted in India suggest that Olyset Plus works against pyrethroid-resistant malaria vectors, but the benefit of the PBO is unclear and needs further investigation in larger studies [50]. However, research from Togo and Burkina Faso found that Olyset Plus performed better than other nets in controlled experiments in which people slept in huts with mosquitoes. Yorkool, a pyrethroid-only net, provides less protection against wild-resistant *An.gambiae* [41,51].

Similar to Olyset Plus, Olyset Duo, a combination of Olyset Net and pyriproxyfen, produces better results on the Benin and Ivory Coast than Olyset Net alone [47,48]. Pyriproxyfen is an insect growth regulator (IGR) that interferes with development and reproduction of malaria vectors, including those resistant to pyrethroids [70,71]. It is particularly effective in preventing malaria vectors from laying their eggs.

Trials conducted in Tanzania demonstrated the promising performance of interceptor nets in both personal protection and blood-feeding inhibition of malaria vectors [43]. Interceptor G2 combines alpha-cypermethrin, a synthetic pyrethroid, with chlorfenapyr, a different insecticide [45,46]. Chlorfenapyr is an insecticide that has a slow-acting mechanism of action. This disrupts the ability of the mosquito to produce energy within its cells. The effectiveness of Interceptor G2 warrants further studies to confirm its long-term benefits [71].

Both Olyset Duo and Interceptor G2 demonstrated the value of combining different insecticides in mosquito nets . The new PermaNet Dual long-lasting insecticidal net (*LLIN*), which contains both chlorfenapyr and deltamethrin insecticides, worked very well against *Anopheles gambiae* vectors. Notably, it outperformed PermaNet 3.0 in all outcome measures evaluated. [42]

Studies on Dawa Plus 3.0 and 4.0 nets showed improved effectiveness due to the addition of PBO [56,57]. Similarly, Veeralin, MAGNet and Miranets performed well against malaria vectors, with veeralin causing higher death rates [59,60,61,62,64]. These findings support the use of new-generation *LLIN*s in areas of high malaria endemicity. Compared with untreated polyester nets, lambda-cyhalothrin-treated ICON® Maxx nets showed significantly improved outcomes.

However, the effectiveness of *LLIN*s can vary, depending on their location. For example, DuraNet worked well in Tanzania but not in Ethiopia [52,53]. Other studies have explored the effectiveness of nets such as LifeNet, Royal Guard, PandaNet 2.0, and Yahe [54,55,65,63]. Some studies have confirmed the good performance of SafeNet NF and SafeNet *LLIN*s by using interceptor nets as a comparator, although more research is needed. [66]

Overall, this review demonstrates the potential of various *LLIN*s in different situations. This highlights the importance of ongoing research, development of new combinations of insecticides, and conducting large-scale trials to create the best strategies for mosquito control [40-66].

Our research showed that OlysetNet, Netprotect, PermaNet2.0, Duranet, and Interceptor performed better than the untreated net (negative control). However, this approach may inflate the apparent efficacy of the candidate nets. We strongly recommend employing a positive control net alongside an untreated negative control to mitigate this bias and obtain more reliable results while also conducting additional Phase II trials focused on diverse ecological settings and various vectors

This work will aid researchers in framing new formulations of concentrations that enhance protection against resistant malaria vectors in specific regions.

## Conclusion

The WHO has set strict standards for testing the effectiveness of *LLIN* in Phase II trials. After 20 washes, these experiments evaluated the efficacy of the nets in eliminating malaria vectors and inhibiting their feeding behavior. Nets that perform well are then considered for further testing in larger studies to determine if people accept them and if they work well in real-world settings.

This review scrutinized *LLIN* testing from various locations to ensure compliance with WHO guidelines, including the washing of the nets and the design of the testing huts. The review found that some nets work well on their own, but adding ingredients, such as PBO and PPF, can significantly improve their effectiveness against malaria vectors that carry diseases. Importantly, these additives are safe, affordable, and easy to use.

This study also highlights that malaria vectors in different areas may respond differently to insecticides. In such situations, the addition of PBO and PPF can significantly boost the effectiveness of *LLIN*s. This information is important for developing robust strategies for controlling mosquito populations.

In summary, this review highlights that incorporating PBO and PPF into *LLIN*s can serve as an effective strategy for combating mosquito-borne diseases, particularly in light of the diverse resistance patterns exhibited by malaria vectors across different regions. These findings can inform the refinement of *LLIN* testing protocols in Phase II trials, ultimately contributing to more effective mosquito control strategies.

## Supporting information

S1 Table Study characteristics table. (DOCX)

S2 Table Experimental hut outcome measures. (DOCX)

S3 Table Checklist. (DOCX)

S1 File Sheet 1: All studies (included and excluded) S1-Sheet 2: Inclusion additional data. S1-Sheet 3: Relative risk data. S1-Sheet 4: Risk of bias data.
(XLSX)

S2 File Reasons for exclusions. (DOCX)

S3 File PRISMA checklist. (DOCX)

## Abbreviations

BFI: Blood Feeding Inhibition
CDC: Centre for Disease Control & Prevention
CTN: Conventional treated nets
CTN: Conventional Treated Nets
D: Deterrence
DDT: Dichloro-diphenyl-trichloroethane
EHT: Experimental Hut Trial
Ex: Exophily
GLP: Good Laboratory Practice
IRS: Indoor Residual Spraying
ITB: Insecticide Treated Bed
ITN: Insecticide-treated nets
ITWL: Insecticide-Treated Wall Linings
k: Number of studies
LLIB: Long Lasting Insecticidal Blankets
LLINs: Long Lasting Insecticidal Nets
MM: Mosquito Mortality
Mo: Mortality
NCVBDCP: National Centre Vector Borne Disease Control Programme
NFME: National Framework for Malarial Elimination
NMCP: National Malaria Control Programme
P.R: Pyrethroid Resistant
P.S: Pyrethroid Susceptible
PBO: Pyrethroid‐Piperonyl Butoxide
PPF: Pyriproxyfen
PPF: Pyriproxyfen
PRISMA: Preferred reporting items for systematic reviews
TDR: Research and Training in Tropical Diseases
UNICEF: United Nations International Children’s Emergency Fund
VBD: Vector-Borne Disease
WHO PQT/VCP: WHO Prequalification Unit/Vector Control Product
WHO: World Health Organization.

## References

[1] Cox FE. History of the discovery of the malaria parasites and their vectors. Parasit Vectors. 2010;3(1):5. doi: 10.1186/1756-3305-3-5 20205846 PMC2825508

[2] World Health Organization. Regional Office for South-East Asia. Anopheline species complexes in South and South-East Asia [Internet]. WHO Regional Office for South-East Asia; 2007 [cited 2022 Oct 20]. Available from: https://apps.who.int/iris/handle/10665/204779

[3] Institute of Medicine (US) Committee on the Economics of Antimalarial Drugs, Arrow KJ, Panosian C, Gelband H. A Brief History of Malaria. Saving Lives, Buying Time: Economics of Malaria Drugs in an Age of Resistance [Internet]. US: National Academies Press; 2004 [cited 2023 Dec 10]. Available from: https://www.ncbi.nlm.nih.gov/books/NBK215638/. 25009879

[4] Nicoletti M. Insect-Borne Diseases in the 21st Century. Academic Press; 2020.

[5] Vector-borne diseases [Internet]. [cited 2023 Dec 10]. Available from: https://www.who.int/news-room/fact-sheets/detail/vector-borne-diseases

[6] van Seventer JM, Hochberg NS. Principles of Infectious Diseases: Transmission, Diagnosis, Prevention, and Control. International Encyclopedia of Public Health. 2017, p. 22–39.

[7] Prugnolle F, Durand P, Ollomo B, Duval L, Ariey F, Arnathau C, et al. A fresh look at the origin of *Plasmodium falciparum*, the most malignant malaria agent. PLoS Pathog. 2011;7(2):e1001283. doi: 10.1371/journal.ppat.1001283 21383971 PMC3044689

[8] Amir A, Cheong FW, de Silva JR, Liew JWK, Lau YL. *Plasmodium knowlesi* malaria: current research perspectives. Infect Drug Resist. 2018;11:1145–55. doi: 10.2147/IDR.S148664 30127631 PMC6089103

[9] Fact sheet about malaria [Internet]. [cited 2023 Nov 30]. Available from: https://www.who.int/news-room/fact-sheets/detail/malaria

[10] World Malaria Report 2023. Available from: https://www.who.int/teams/global-malaria-programme/reports/world-malaria-report-2023

[11] Malaria– India’s Battle Against a Complex Disease [Internet]. World Bank. [cited 2023 Dec 10]. Available from: https://www.worldbank.org/en/news/feature/2010/04/23/malaria-indias-battle-against-a-complex-disease

[12] Operational Manual for Malaria Elimination in India 2016. (Version 1) [Internet]. [cited 2023 Dec 21]. Available from: https://ncvbdc.mohfw.gov.in/WriteReadData/l892s/5232542721532941542.pdf

[13] Kamareddine L. The biological control of the malaria vector. Toxins (Basel). 2012;4(9):748–67. doi: 10.3390/toxins4090748 23105979 PMC3475227

[14] Bratati B. JaypeeDigital | National Vector Borne Disease Control Programme—Malaria [Internet]. [cited 2022 Oct 20]. Available from: https://www.jaypeedigital.com/book/9789386322722/chapter/ch13

[15] WHO certifies Azerbaijan and Tajikistan as malaria-free [Internet]. [cited 2023 Nov 30]. Available from: https://www.who.int/news/item/29-03-2023-who-certifies-azerbaijan-and-tajikistan-as-malaria-free

[16] Countries and territories certified malaria-free by WHO [Internet]. [cited 2023 Dec 10]. Available from: https://www.who.int/teams/global-malaria-programme/elimination/countries-and-territories-certified-malaria-free-by-who

[17] CDC-Centers for Disease Control and Prevention. CDC - Malaria - Malaria Worldwide - Impact of Malaria [Internet]. 2021 [cited 2022 Oct 20]. Available from: https://www.cdc.gov/malaria/malaria_worldwide/impact.html

[18] Lengeler C. Insecticide-treated bed nets and curtains for preventing malaria. The Cochrane database of systematic reviews [Internet]. 2004 [cited 2022 Oct 20]; Available from: https://pubmed.ncbi.nlm.nih.gov/15106149/. 10.1002/14651858.CD000363.pub2 15106149

[19] Dhiman S, Veer V. Culminating anti-malaria efforts at long lasting insecticidal net?. J Infect Public Health. 2014;7(6):457–64. doi: 10.1016/j.jiph.2014.06.002 25092624

[20] Kenney CS. A Case Study in Fundraising, Logistics, and Collective Action for a Neglected Disease | Policy Commons. [cited 2024 Sep 11]; Available from: https://policycommons.net/artifacts/9768427/a-case-study-in-fundraising-logistics-and-collective-action-for-a-neglected-disease/10657675/.

[21] Miller JM, Korenromp EL, Nahlen BL, W Steketee R. Estimating the number of insecticide-treated nets required by African households to reach continent-wide malaria coverage targets. JAMA. 2007;297(20):2241–50. doi: 10.1001/jama.297.20.2241 17519414

[22] Sadasivaiah S, Tozan Y, Breman JG. Dichlorodiphenyltrichloroethane (DDT) for indoor residual spraying in Africa: How can it be used for malaria control? In: Defining and Defeating the Intolerable Burden of Malaria III: Progress and Perspectives: Supplement to Volume 77(6) of *American Journal of Tropical Medicine and Hygiene*. American Society of Tropical Medicine and Hygiene; 2007 [cited 2022 Oct 20]. Available from: https://www.ncbi.nlm.nih.gov/books/NBK1724/. 18165500

[23] Kilian A, Byamukama W, Pigeon O, Atieli F, Duchon S, Phan C. Long-term field performance of a polyester-based long-lasting insecticidal mosquito net in rural Uganda. Malar J. 2008;7 49. doi: 10.1186/1475-2875-7-49 18355408 PMC2330059

[24] N’Guessan R, Darriet F, Doannio JM, Chandre F, Carnevale P. Olyset Net efficacy against pyrethroid-resistant *Anopheles gambiae* and *Culex quinquefasciatus* after 3 years’ field use in C te d’Ivoire. Med Vet Entomol. 2001;15(1):97–104. doi: 10.1046/j.1365-2915.2001.00284.x 11297108

[25] UNICEF. Fighting malaria with long-lasting insecticidal nets (LLINs) | UNICEF Supply Division [Internet]. [cited 2022 Oct 20]. Available from: https://www.unicef.org/supply/stories/fighting-malaria-long-lasting-insecticidal-nets-llins

[26] WHO. Report of the fifth WHOPES working group meeting: WHO/HQ, Geneva, 30-31 October 2001 [Internet]. [cited 2022 Oct 20]. Available from: https://www.who.int/publications-detail-redirect/who-cds-whopes-2001.4

[27] Report of the seventh WHOPES working group meeting WHO/HQ, Geneva 2–4 December 2003 [Internet]. [cited 2022 Oct 20]. Available from: https://www.who.int/publications-detail-redirect/who-cds-whopes-2004.8

[28] World Health Organization. Report of the tenth WHOPES working group meeting: WHO/HQ, Geneva, 11-14 December 2006: review of: Spinosad 0.5% GR & 12% SC, Lambda-cyhalothrin 10% CS, K-O Tab 1-2-3, Interceptor [Internet]. World Health Organization; 2007. Report No.: WHO/CDS/NTD/WHOPES/2007.1. Available from: https://apps.who.int/iris/handle/10665/69465

[29] WHO Pesticide Evaluation Scheme. Working Group. Meeting (11th: 2007: Geneva S, Organization WH, Scheme WPE. Report of the eleventh WHOPES working group meeting: WHO/HQ, Geneva, 10-13 December 2006: review of spinosad 7.48% DT, netprotect, duranet, dawaplus, Icon Maxx [Internet]. World Health Organization; 2008. Report No.: WHO/HTM/NTD/WHOPES/2008.1. Available from: https://apps.who.int/iris/handle/10665/69732

[30] World Health Organization. Report of the twelfth WHOPES working group meeting, WHO/HQ, Geneva, 8-11 December 2008: review of bioflash GR, permanet 2.0, permanet 3.0, permanet 2.5, lambda-cyhalothrin LN [Internet]. World Health Organization; 2009. Report No.: WHO/HTM/NTD/WHOPES/2009.1. Available from: https://apps.who.int/iris/handle/10665/69986

[31] World Health Organization. Report of the thirteenth [13th] WHOPES working group meeting: WHO/HQ, Geneva, 28-30 July 2009: review of Olyset LN, Dawaplus 2.0 LN, Tianjin Yorkool LN [Internet]. World Health Organization; 2009. Report No.: WHO/HTM/NTD/WHOPES/2009.5. Available from: https://apps.who.int/iris/handle/10665/44212

[32] World Health Organization. Report of the fourteenth [14th] WHOPES working group meeting: WHO/HQ, Geneva, 11-15 April2011: review of Spinosad EC, Lifenet LN, Magnet LN, Royal Sentry LN, Yahe LN [Internet]. World Health Organization; 2011. Report No.: WHO/HTM/NTD/WHOPES/2011.7. Available from: https://apps.who.int/iris/handle/10665/44669

[33] World Health Organization, WHO Pesticide Evaluation Scheme. Working Group. Meeting 15th 2012 G. Report of the fifteenth WHOPES working group meeting: WHO/HQ, Geneva, 18-22 June 2012: review of Olyset plus, Interceptor LN, Malathion 440 EW, Vectobac GR [Internet]. World Health Organization; 2012 [cited 2022 Oct 20]. Available from: https://apps.who.int/iris/handle/10665/75304

[34] World Health Organization, WHO Pesticide Evaluation Scheme. Working Group. Meeting (16th: 2013: Geneva S. Report of the sixteenth WHOPES working group meeting: WHO/HQ, Geneva, 22-30 July 2013: review of Pirimiphos-methyl 300 CS, Chlorfenapyr 240 SC, Deltamethrin 62.5 SC-PE, Duranet LN, Netprotect LN, Yahe LN, Spinosad 83.3 Monolayer DT, Spinosad 25 Extended release GR [Internet]. World Health Organization; 2013 [cited 2022 Oct 20]. Available from: https://apps.who.int/iris/handle/10665/90976

[35] WHO. Report of the eighteenth WHOPES working group meeting: WHO/HQ, Geneva, 29 June−1 July 2015 [Internet]. [cited 2022 Oct 20]. Available from: https://www.who.int/publications-detail-redirect/9789241509428

[36] World Health Organization. Report of the nineteenth WHOPES working group meeting: WHO/HQ, Geneva, 8-11 February 2016: review of Veeralin LN, VectoMax GR, Bactivec SC [Internet]. World Health Organization; 2016 [cited 2022 Oct 20]. Available from: https://apps.who.int/iris/handle/10665/205588

[37] World Health Organization. Report of the twentieth WHOPES working group meeting, WHO/HQ, Geneva, 20–24 March 2017 [Internet]. World Health Organization; 2017. Report No.: WHO/HTM/NTD/WHOPES/2017.04. Available from: https://apps.who.int/iris/handle/10665/258921

[38] Institute EPH. Protocol for Evaluation of the Efficacy of Long-Lasting Insecticide Treated Nets Under Field Conditions in Ethiopia, 2017. 2017 [cited 2024 Sep 11]; Available from: http://repository.iphce.org/xmlui/handle/123456789/468

[39] Guidelines for laboratory and field testing of long-lasting insecticidal nets [Internet]. [cited 2024 Sep 11]. Available from: https://www.who.int/publications/i/item/9789241505277

[40] Tungu P, Magesa S, Maxwell C, Malima R, Masue D, Sudi W, et al. Evaluation of PermaNet 3.0 a deltamethrin-PBO combination net against *Anopheles gambiae* and pyrethroid resistant *Culex quinquefasciatus* mosquitoes: an experimental hut trial in Tanzania. Malar J. 2010;9:21. doi: 10.1186/1475-2875-9-21 20085631 PMC2817703

[41] Ketoh GK, Ahadji-Dabla KM, Chabi J, Amoudji AD, Apetogbo GY, Awokou F, et al. Efficacy of two PBO long lasting insecticidal nets against natural populations of *Anopheles gambiae* s.l. in experimental huts, Kolokopé, Togo. PLoS One. 2018;13(7):e0192492. doi: 10.1371/journal.pone.0192492 29995894 PMC6040683

[42] Zahouli JZB, Edi CAV, Yao LA, Lisro EG, Adou M, Koné I, et al. Small-scale field evaluation of PermaNet® Dual (a long-lasting net coated with a mixture of chlorfenapyr and deltamethrin) against pyrethroid-resistant *Anopheles gambiae* mosquitoes from Tiassalé, Côte d’Ivoire. Malar J. 2023;22(1):36. doi: 10.1186/s12936-023-04455-z 36726160 PMC9893697

[43] Malima R, Tungu PK, Mwingira V, Maxwell C, Magesa SM, Kaur H, et al. Evaluation of the long-lasting insecticidal net Interceptor LN: laboratory and experimental hut studies against anopheline and culicine mosquitoes in northeastern Tanzania. Parasit Vectors. 2013;6(1):296. doi: 10.1186/1756-3305-6-296 24499488 PMC4028879

[44] Gunasekaran K, Sahu SS, Vijayakumar T, Vaidyanathan K, Yadav RS, Pigeon O, et al. Comparison of efficacy of five types of long-lasting insecticidal nets against *Anopheles fluviatilis*, the primary malaria vector in east-central India. J Med Entomol. 2014;51(4):785–94. doi: 10.1603/me13136 25118410

[45] Camara S, Ahoua Alou LP, Koffi AA, Clegban YCM, Kabran J-P, Koffi FM, et al. Efficacy of Interceptor® G2, a new long-lasting insecticidal net against wild pyrethroid-resistant *Anopheles gambiae* s.s. from Côte d’Ivoire: a semi-field trial. Parasite. 2018;25:42. doi: 10.1051/parasite/2018042 30088473 PMC6082037

[46] Tungu PK, Michael E, Sudi W, Kisinza WW, Rowland M. Efficacy of interceptor® G2, a long-lasting insecticide mixture net treated with chlorfenapyr and alpha-cypermethrin against *Anopheles funestus*: experimental hut trials in north-eastern Tanzania. Malar J. 2021;20(1):180. doi: 10.1186/s12936-021-03716-z 33836778 PMC8033724

[47] Ngufor C, N’guessan R, Fagbohoun J, Odjo A, Malone D, Akogbeto M, et al. Olyset Duo® (a pyriproxyfen and permethrin mixture net): an experimental hut trial against pyrethroid resistant *Anopheles gambiae* and *Culex quinquefasciatus* in Southern Benin. PLoS One. 2014;9(4):e93603. doi: 10.1371/journal.pone.0093603 24699827 PMC3974762

[48] Koffi AA, Ahoua Alou LP, Djenontin A, Kabran J-PK, Dosso Y, Kone A, et al. Efficacy of Olyset® Duo, a permethrin and pyriproxyfen mixture net against wild pyrethroid-resistant *Anopheles gambiae* s.s. from Côte d’Ivoire: an experimental hut trial. Parasite. 2015;22 28. doi: 10.1051/parasite/2015028 26489480 PMC4613874

[49] Pennetier C, Bouraima A, Chandre F, Piameu M, Etang J, Rossignol M, et al. Efficacy of Olyset® Plus, a new long-lasting insecticidal net incorporating permethrin and piperonyl-butoxide against multi-resistant malaria vectors [corrected]. PLoS One. 2013;8(10):e75134. doi: 10.1371/journal.pone.0075134 24116029 PMC3792972

[50] Gunasekaran K, Sahu SS, Vijayakumar T, Subramanian S, Yadav RS, Pigeon O, et al. An experimental hut evaluation of Olyset Plus, a long-lasting insecticidal net treated with a mixture of permethrin and piperonyl butoxide, against *Anopheles fluviatilis* in Odisha State, India. Malar J. 2016;15(1):375. doi: 10.1186/s12936-016-1424-1 27439398 PMC4955161

[51] Toe KH, Müller P, Badolo A, Traore A, Sagnon N, Dabiré RK, et al. Do bednets including piperonyl butoxide offer additional protection against populations of *Anopheles gambiae* s.l. that are highly resistant to pyrethroids? An experimental hut evaluation in Burkina Fasov. Med Vet Entomol. 2018;32(4):407–16. doi: 10.1111/mve.12316 29998497

[52] Mahande AM, Msangi S, Lyaruu LJ, Kweka EJ. Bio-efficacy of DuraNet® long-lasting insecticidal nets against wild populations of *Anopheles arabiensis* in experimental huts. Trop Med Health. 2018;46:36. doi: 10.1186/s41182-018-0118-5 30410416 PMC6219078

[53] Gebremariam B, Birke W, Zeine W, Ambelu A, Yewhalaw D. Evaluation of long-lasting insecticidal nets (DuraNet®) under laboratory and semi-field conditions using experimental huts against anopheles mosquitoes in Jimma Zone, Southwestern Ethiopia. Environ Health Insights. 2021;15 1178630220974730. doi: 10.1177/1178630220974730 33488090 PMC7809299

[54] Gunasekaran K, Sahu SS, Vijayakumar T, Subramanian S, Jambulingam P. Bio-efficacy of LifeNet, a deltamethrin incorporated long-lasting insecticidal net, as assessed in experimental huts against *Anopheles fluviatilis*, a major malaria vector in east-central India. Acta Trop. 2018;187:151–7. doi: 10.1016/j.actatropica.2018.08.004 30092223

[55] Djènontin A, Moiroux N, Bouraïma A. et al. Field efficacy of a new deltamethrin long lasting insecticidal net (LifeNet©) against wild pyrethroid-resistant *Anopheles gambiae* in Benin. BMC Public Health 2018;18:947. doi: 10.1186/s12889-018-5876-9 30068334 PMC6090760

[56] Bayili K, N’Do S, Yadav RS, Namountougou M, Ouattara A, Dabiré RK, et al. Experimental hut evaluation of DawaPlus 3.0 LN and DawaPlus 4.0 LN treated with deltamethrin and PBO against free-flying populations of *Anopheles gambiae* s.l. in Vallée du Kou, Burkina Faso. PLoS One. 2019;14(12):e0226191. doi: 10.1371/journal.pone.0226191 31869350 PMC6927612

[57] Gunasekaran K, Sahu SS, Vijayakumar T, Subramanian S, Rahi M, Jambulingam P. Evaluation of DawaPlus 3.0 and DawaPlus 4.0, deltamethrin-PBO combination nets against pyrethroid-resistant *Anopheles culicifacies* in experimental huts in India. Malar J. 2020;19(1):43. doi: 10.1186/s12936-020-3119-x 31973734 PMC6979062

[58] Tungu PK, Malima R, Mosha FW, Lyimo I, Maxwell C, Kaur H, et al. Evaluation of ICON Maxx, a long-lasting treatment kit for mosquito nets: experimental hut trials against anopheline mosquitoes in Tanzania. Malar J. 2015;14:225. doi: 10.1186/s12936-015-0742-z 26025026 PMC4487969

[59] Kasinathan G, Sahu SS, Tharmalingam V, Swaminathan S, Rahi M, Purushothaman J. Evaluation of MAGNet, a long-lasting insecticidal mosquito net against *Anopheles fluviatilis* in experimental huts in India. Malar J. 2019;18(1):59. doi: 10.1186/s12936-019-2692-3 30841885 PMC6404338

[60] Kweka EJ, Tungu PK, Mahande AM, Mazigo HD, Sayumwe S, Msangi S, et al. Bio-efficacy and wash resistance of MAGNet long-lasting insecticidal net against wild populations of *Anopheles funestus* in experimental huts in Muheza, Tanzania. Malar J. 2019;18(1):335. doi: 10.1186/s12936-019-2973-x 31570107 PMC6771101

[61] Oumbouke WA, Koffi AA, Alou LPA, Rowland M, N’Guessan R. Evaluation of standard pyrethroid based LNs (MiraNet and MagNet) in experimental huts against pyrethroid resistant *Anopheles gambiae* s.l. M’bé, Côte d’Ivoire: Potential for impact on vectorial capacity. PLoS One. 2019;14(4):e0215074. doi: 10.1371/journal.pone.0215074 30973948 PMC6459542

[62] Kasinathan G, Sahu SS, Krishnamoorthy N, Baig MM, Thankachy S, Dash S, et al. Efficacy evaluation of Veeralin LN, a PBO-incorporated alpha-cypermethrin long-lasting insecticidal net against *Anopheles culicifacies* in experimental huts in Odisha State. Malar J. 2020;19(1):402. doi: 10.1186/s12936-020-03480-6 33172495 PMC7654164

[63] Clegban C-MY, Camara S, Koffi AA, Ahoua Alou LP, Kabran Kouame J-P, Koffi AF, et al. Evaluation of Yahe® and Panda® 2.0 long-lasting insecticidal nets against wild pyrethroid-resistant *Anopheles gambiae* s.l. from Côte d’Ivoire: an experimental hut trial. Parasit Vectors. 2021;14(1):347. doi: 10.1186/s13071-021-04843-x 34210362 PMC8247218

[64] Tungu PK, Waweru J, Karthi S, Wangai J, Kweka EJ. Field evaluation of Veeralin, an alpha-cypermethrin + PBO long-lasting insecticidal net, against natural populations of *Anopheles funestus* in experimental huts in Muheza, Tanzania. Curr Res Parasitol Vector Borne Dis. 2021;1:100030. doi: 10.1016/j.crpvbd.2021.100030 35284898 PMC8906063

[65] Ngufor C, Agbevo A, Fagbohoun J, Fongnikin A, Rowland M. Efficacy of Royal Guard, a new alpha-cypermethrin and pyriproxyfen treated mosquito net, against pyrethroid-resistant malaria vectors. Sci Rep. 2020;10(1):12227. doi: 10.1038/s41598-020-69109-5 32699237 PMC7376134

[66] Azizi S, Snetselaar J, Kaaya R, Matowo J, Onen H, Shayo M, et al. Implementing OECD GLP principles for the evaluation of novel vector control tools: a case study with two novel LLINs, SafeNet® and SafeNet NF®. Malar J. 2022;21(1):183. doi: 10.1186/s12936-022-04208-4 35690824 PMC9188019

[67] McGuinness LA, Higgins JPT. Risk-of-bias VISualization (robvis): An R package and Shiny web app for visualizing risk-of-bias assessments. Res Synth Methods. 2021;12(1):55–61. doi: 10.1002/jrsm.1411 32336025

[68] Bingham G, Strode C, Tran L, Khoa PT, Jamet HP. Can piperonyl butoxide enhance the efficacy of pyrethroids against pyrethroid-resistant *Aedes aegypti*? Trop Med Int Health. 2011;16(4):492–500. doi: 10.1111/j.1365-3156.2010.02717.x 21324051

[69] Ohashi K, Nakada K, Ishiwatari T, Miyaguchi J, Shono Y, Lucas JR, et al. Efficacy of pyriproxyfen-treated nets in sterilizing and shortening the longevity of *Anopheles gambiae* (Diptera: Culicidae). J Med Entomol. 2012;49(5):1052–8. doi: 10.1603/me12006 23025186

[70] Dhadialla TS, Carlson GR, Le DP. New insecticides with ecdysteroidal and juvenile hormone activity. Annu Rev Entomol. 1998;43 545–69. doi: 10.1146/annurev.ento.43.1.545 9444757

[71] Raghavendra K, Barik TK, Sharma P, Bhatt RM, Srivastava HC, Sreehari U, et al. Chlorfenapyr: a new insecticide with novel mode of action can control pyrethroid resistant malaria vectors. Malar J. 2011;10:16. doi: 10.1186/1475-2875-10-16 21266037 PMC3039634

