# Supplementary material for: Evaluation of long lasting insecticidal nets in experimental huts and WHO PQT/VCP compliance: A systematic review
Source: PLoS One. 2025 Mar 12;20(3):e0318673. doi: 10.1371/journal.pone.0318673 (PMC11902051; doi:10.1371/journal.pone.0318673)
Supplement: S1 Table — (DOCX) [file pone.0318673.s001.docx]

**Table S1: Study characteristics and summary of included studies assessing the efficacy of various LLINS.**

| **Author &Year** | **Place of Evaluation** | **Candidate Net / Fabric / Insecticide Used** | **Comparator Net** | **Testing method** | **Vector species** | **% of mortality in the testing method** | **Remarks** |
| --- | --- | --- | --- | --- | --- | --- | --- |
| Tungu,*et al.* (2010) | Tanzania | - PermaNet 3.0–   **Polyethylene** (Top Panel): 4g/kg Deltamethrin & 25g/kg Piperonyl butoxide for 100 denier.  **Polyester (**Side Panel): 2.8g/kg Deltamethrin for 75 denier. | - PermaNet2.0 – **Polyester**: 1.8g/kg Deltamethrin for 75 denier. - CTN- **Polyester** 25 mg/m^2^ deltamethrin for 100 deniers. - Untreated **Polyester** Net. | Cone bioassay | *An. gambiae* Kisumu (Susceptible strain) | PermaNet 3.0: 100%  PermaNet 2.0: 98%  CTN- Polyester:  8% | After 20 washes both PermaNet products have high rates of mortality against *An. gambiae.*[40] |
| Malima, *et al.* (2013) | Tanzania | - Interceptor -   **Polyester**- 6.7 g AI/kg Alpha-cypermethrin for 75 denier. | - CTN- **Polyester**   200 mg/m^2^ alpha-cypermethrin.   - Untreated **Polyester** Nets | Cone bioassay. | *An. gambiae* (Susceptible strain) | Interceptor® LN :100%  CTN:68% | Interceptor outperformed the CTN in terms of personal protection and Blood feeding inhibition. This LN meets WHO Criteria.[41] |
| Pennetier *et al.* (2013) | Benin | - Olyset Plus -   **Polyethylene** Permethrin 2% w/w & Piperonyl butoxide 10 g/kg for 150 denier. | - Olyset Net – **Polyethylene**   Permethrin 2%w/w   - CTN-**Polyester** 500 mg AI/m^2^ Permethrin - Untreated  **Polyethylene** net | Cone bioassay | *An. gambiae* (Resistant strain) | Olyset Plus:64% | The benefit of incorporating PBO was seen by exhibiting higher blood feeding inhibition (i.e., personal protection). However further evaluation is needed in large-scale field trail for assessing the durability and acceptability.[42] |
| Gunasekaran, *et al.* (2014) | India | - Olyset - **Polyethylene**   1,000mg/m^2^ Permethrin.   - Netprotect-**Polyethylene**   63mg/m^2^ Deltamethrin   - PermaNet 2.0- **Polyester**, 55mg/m^2^Deltamethrin - DuraNet- **Polyethylene**   221mg/m^2^Alphacypermethrin.   - Interceptor- **Polyester**, Alphacypermethrin 200mg/m^2^ | Untreated Net: 4% | Cone bioassay | *An. fluviatilis* (Susceptible strain) | - Olyset: 98% - Netprotect: 100% - PermaNet 2.0: 100% - DuraNet:   100%   - Interceptor: 98% - Untreated Net:4% | All three synthetic pyrethroid nets exhibits almost similar outcomes but they differ in killing effect. Alphacypermethrin nets treated LNs killed greater number of mosquitoes that entered the huts. [43] |
| Ngufor C *et al.*  (2014) | Southern  Benin | - Olyset Net Duo ®- **Polyethylene**+ Permethrin (2% w/w) & Pyriproxyfen (1% w/w) | - Olyset Net ® - Pyriproxyfen (PPF) LN - Untreated **Polyethylene** Net | Tunnel Test | *An. gambiae*  (Resistant strain) | - Olyset Net Duo: 100 - Olyset Net:91 - Pyriproxyfen:5 - Untreated Net:0 | Shows efficacy against Pyrethroid resistant *An. gambiae* when compared with Olyset Net.[44] |
| Koffi *et al.*  (2015) | Côte d’Ivoire | - Olyset Net® Duo-   **Polyethylene,** 2% (w/w) Permethrin & 1% (w/w) Pyriproxyfen | - Olyset Net*:*  **Polyethylene** 2% (w/w) Permethrin. - Pyriproxyfen 1% (w/w) Treated **Polyethylene** Net. - Untreated **Polyethylene** Net | Cone bioassay | *An. gambiae*  (Resistant strain) | - Olyset Net Duo-:78 - Olyset Net:13 - PPF Net:0 - Untreated Net:2 | After washing and field trails Olyset Net® Duo performed much better than Olyset Net but this LLIN did not impact the fecundity rates of the wild pyrethroid resistant *An. gambiae*. in addition to that *kdr* have metabolic resistance. So, impact PPF needs to be further investigation[45] |
| Tungu *et al.* (2015) | Tanzania | - ICON Maxx –   **Polyester** 55 mg AI/m^2^ Lambda-cyhalothrin | - CTN-   **Polyester**  15 mg/m^2^ lambda-cyhalothrin.   - Untreated **Polyester** Net | Cone-bioassay | *An. funestus* (Susceptible strain) | ICON Maxx :92  CTN:12 | This LLIN performs significantly much better than other nets in terms of mortality and outcome measures.[46] |
| Gunasekaran, *et al.*(2016) | India | - Olyset Plus   **Polyethylene**,  Permethrin 2 % w/w & Piperonyl butoxide 1 % w/w | - Olyset Net- **Polyethylene** Permethrin 2% w/w - CTN-**Polyester** 500mg/m^2^ - Untreated **Polyester** Net | Cone-bioassay | *An. fluviatilis*  (Susceptible strain) | Olyset Plus:100  Olyset Net:100  CTN:96  Untreated:4 | Olyset Plus net performance is similar with Olyset Net and CTN could be effective against susceptible vectors. But the benefit of incorporating PBO is not demonstrated. So, this study needs to be further evaluated before phase III for community acceptance.[47] |
| Ketoh *et al.* 2018 | Kolokope´Togo | - PermaNet 3.0–   **Polyethylene** (Top Panel): 4g/kg Deltamethrin & 25g/kg Piperonyl butoxide for 100 denier.  **Polyester (**Side Panel): 2.8g/kg Deltamethrin for 75 denier.   - Olyset Plus-   **Polyethylene** -Permethrin & Piperonyl butoxide   - Yorkool-   **Polyester**, Deltamethrin | - Untreated Net | Cone bioassay | *An. gambiae*  (Susceptible strain) | PermaNet 3.0 -100  Olyset plus -98.3  Yorkool - 96  Untreated Net:0 | PermaNet 3.0 and Olyset plus shows better performance and can provide additional protection in terms of reduction in blood feeding and increase in mosquito mortality, compared to a pyrethroid-only net i.e.,yorkool against wild resistant *An.gambiae s.l* [48] |
| Gunasekaran *et.al* (2018) | East-central India | - LifeNet-   **Polypropylene**-340 mg AI/m^2^ Deltamethrin for 100 denier. | - CTN-   **Polyester** 25 mg/m^2^ deltamethrin.   - Untreated **Polypropylene** Net | Cone bioassay | *An. fluviatilis*  (Susceptible strain) | LifeNet **-** 100  CTN:56  Untreated Net: 4 | In terms of outcome measures in experimental huts Life Net met WHO Criteria.[49] |
| Toe KH *et al.* (2018) | Burkina  Fasov | - Olyset Plus- **Polyethylene**, 8.6 × 10 −4 kg/m ^2^ Permethrin & 4.3 × 10 −4 kg/m ^2^ Piperonyl butoxide - PermaNet 3.0 – **Polyethylene** (Top Panel): 4g/kg Deltamethrin & 25g/kg Piperonyl butoxide for 100 denier.   **Polyester** (Side Panel): 2.8g/kg Deltamethrin | - Olyset Net – **polyethylene**, 8.6 × 10−4 kg/m ^2^ permethrin. - PermaNet 2.0-   **Polyester**, 5.5 × 10 −5 kg/m ^2^ Deltamethrin.   - Untreated Net - Dawa Plus 2.0-   **Polyester**,8.0 × 10 −5 kg/m^2^ Deltamethrin. | Cone bioassay | *An. gambiae Kisumu*  Susceptibile strain) | Olyset® Net: 94.90  Olyset® Plus: 99.21  PermaNet ®2.0:  97.95  PermaNet® 3.0:  100 | Mortality rates are higher with PBO LNs these nets may be effective against malaria vectors.[50] |
| Mahande, *et al.* (2018) | Tanzania | - DuraNet **Polyethylene** -5.8 g/kg Alphacypermethrin | - PermaNet® 3.0 - Untreated **polystyrene** net | Cone-bioassay | *An. arabiensis*  (Resistant strain) | DuraNet 100  PermaNet:100 | Duranet met WHO Criteria by exhibiting efficacy over wild population of *An. arabiensis.* [51] |
| Camara *et al.* (2018) | Côte d’Ivoire | - Interceptor G2   **Polyester**-100 mg/m^2^ Alpha-cypermethrin & 200 mg/m^2^ chlorfenapyr. | - Interceptor® -**Polyester**- 200 mg/m^2^ alpha-cypermethrin - CTN- Polyester- 200 mg/m^2^ chlorfenapyr. - Untreated polyester Nets | Cone bioassay | *An. gambiae*  (Resistant strain) | Interceptor G2:100%  Interceptor:100  CTN:66  Untreated Net:3 | After 20 times washing and field trails mortality rates are higher in Interceptor G2 and met the WHO criteria to undergo Phase III trials.[52] |
| Djènontin  *et al.*(2018) | Benin | - LifeNet- **Polypropylene**-340 mg AI/m2 Deltamethrin for 100 denier. | - CTN-   **Polyester** 25 mg/m^2^ deltamethrin.   - Untreated **Polypropylene** Net | Cone bioassay | *An. gambiae*  (Resistant strain) | Life Net:100  CTN:60  Untreated Net:0 | LifeNet fulfills the WHO Criteria and give equal or better performance when compared to CTNs.[53] |
| Kasinathan, *et al.*(2019) | India | - MAGNet- High-density **polyethylene**-Alpha-cypermethrin for 150 denier. | - Dura Net - Untreated Polyester Net | Cone bioassay. | *An. fluviatilis* (Susceptible strain) | MAGNet-100  DuraNet:100  Untreated Net:0 | MAGNet exhibits 100% mortality after washing 25 times and met WHO Criteria. Both nets performed similar in terms of personal protection, Exit rates and overall killing effect .[54] |
| Bayili *et al.* (2019) | Burkina Faso (West Africa) | - DawaPlus 3.0-   **Polyethylene** (Top Panel): 120 mg ai/m^2^ Deltamethrin & 440 mg/m^2^PBO for 130 denier.  **Polyester** (Side Panel): 100 mg/m^2^Deltamethrin for 100 denier.   - DawaPlus 4.0-   **Polyethylene** 120 mg ai/m^2^Deltamethrin & 440 mg/m^2^ Piperonyl butoxide for 130 denier. | - DawaPlus 2.0 **Polyester** -80 mg/a.i m^2^ deltamethrin. - Untreated **Polyester** Net. | Cone bio-assay | *An. gambiae s.l* (Susceptible strain) | DawaPlus 3.0- 100%  DawaPlus 4.0- 100%  Dawa Plus 2.0: 99  Untreated:0 | Both the Nets met the WHOPES Phase II efficacy requirements for LLINs. Against wild resistant strain mortality decreased to 60% and 50% in both nets.[55] |
| Kweka, *et al*. (2019) | Tanzania | - MAGNet   **Polyethylene**-4.35–7.25 g AI/kg Alpha-cypermethrin | - DuraNet - Interceptor - Untreated Polyester Net. | Cone-bioaasay | *An. funestus.*  (Resistant strain) | MAGNet-99.2  DuraNet: 98 | MagNet shows similar outcome measure to the standard approved DuraNet thus met the WHO criteria.[56] |
| Oumbouke WA, *et.al* (2019) | Côte  d’Ivoire | - MiraNet- High-density **polyethylene** 4.5g/kg Alpha-cypermethrin for 135 denier. - MagNet- High-density **polyethylene**   5.8g/kg Alpha-cypermethrin for 150 denier. | Untreated  **Polyester** Net | Cone-bioassay | *An. gambiae*  (Susceptible strain) | MiraNet: > 99%  MagNet:> 99%  Untreated Net:0 | Despite of high resistance intensity both nets show appreciable protection against mosquito bites and induce slightly greater mortality.[57] |
| Gunasekaran, *et al.* (2020) | India | - DawaPlus 3.0 –   **Polyethylene** *Top Panel*: 120 mg ai/m^2^ Deltamethrin & 480 mg/m^2^ Piperonyl butoxide  **Polyester** *Side Panel*: 105 mg/m^2^Deltamethrin   - DawaPlus 4.0 – **Polyethylene**   120 mg ai/m^2^ Deltamethrin & 480 mg/m^2^Piperonyl butoxide. | - DawaPlus 2.0 **Polyester** -80 mg/a.i m^2^ deltamethrin. - Untreated **Polyester** Net. | Cone-bioassay | *An. culicifacies*  (Resistant strain) | DawaPlus 3.0 :70  DawaPlus 4.0 :36  DawaPlus 2.0:34 | After 20 washes in cone bioassay and hut trails these both nets may not be as effective as required to control the resistant vector.[58] |
| Ngufor *et al* (2020) | South Benin | - Royal Guard- High-density polyethylene (HDPE) and Linear low-density polyethylene (LLDPE) + Alpha-cypermethrin and Pyriproxyfen 5.5 g/kg (225 ± 56.5 mg/m^2^) | - Royal Sentry - DuraNet - Pyriproxyfen Net - Untreated polyethylene Net | Cone-bioassay | *An. gambiae*  (Susceptible strain) | Royal Guard:83%  Royal Sentry:90 | Royal Guard met the WHO criteria. The effect of Pyriproxyfen is 100% compared with Royal Sentry (67%) after 20 washes. [59] |
| Kasinathan, *et al.* (2020) | India | - Veeralin®-   **Polyethylene,** 6.0 ​ ​± 25% g/kg Alpha-cypermethrin & 2.2 ± ​25% g/kg Piperonyl butoxide. | - MagNet - Untreated Polyester Net | Cone-bioaasay | *An. culicifacies*  (Resistant strain) | Veeralin: 76  MagNet:78  Untreated Net:0 | Both Veeralin performed better than MAGNet in terms of overall killing effect and exit rates. This proves that LLIN is an efficient tool against pyrethroid resistant vectors.[60] |
| Clegban, *et al.* (2021) | Côte d’Ivoire  (West Africa) | - Yahe LN –   **Polyester**,1.85 g AI/kg Deltamethrin for 75 denier   - Panda Net 2.0- **Polyethylene,**1.8 g AI/kg Deltamethrin for 110 denier | - PermaNet 2.0. - CTNI: Polyethylene - CTN2: Polyester - Untreated Polyester Net | Cone bioassay. | *An. gambiae s.l* (Susceptible strain) | Yahe LN: 90.6%  Panda Net 2.0: 96.6%  CTN1:90  CTN2:99  PermaNet 2.0:86.5  Untreated Net:0 | Both Yahe® LN and Panda®LN met the WHO Criteria by exhibiting effective personal protection against mosquito bites. But for pyrethroid resistant strains none of these nets reached the WHO criteria. [61] |
| Tungu *et.al.* (2021) | Tanzania | - Veeralin-   **Polyethylene**- 6.0 ​ ​± 25% g/kg Alpha-cypermethrin & 2.2 ± ​25% g/kg Piperonyl butoxide. | - PermaNet 3.0   **Polyethylene** (Top Panel): 4g/kg Deltamethrin & 25g/kg Piperonyl butoxide for 100 denier.  **Polyester** (Side Panel): 2.8g/kg Deltamethrin for 75 denier.   - DuraNet   **Polyethylene** -5.8 ± 25% g/kg Alphacypermethrin   - Untreated **Polyester** Net | Cone-bioaasay | *An. gambiae* and *An. funestus s.l.*  (Resistant strain) | Veeralin:100  PermaNet 3.0:100  DuraNet:100  Untreated Net:0 | Veeralin performs similarly with PermaNet 3.0 against malaria vectors after washing 20 times before hut trails. This provides evidence that Veeralin LLIN has met WHOPES mortality criteria for LLIN.[62] |
| Tungu, *et al.* (2021) | Tanzania | - Interceptor® G2 – Polyester   Alpha-cypermethrin + chlorfenapyr | - Interceptor G1 LN - CTN-Chlorfenapyr - Untreated polyester Net | Cone-bioassay | *An. gambiae* (Susceptible strain) | Interceptor G2:86  Interceptor G1:100  CTN:90 | Mortality rates of Interceptor G2 in two trials met the WHO criteria but there is no entomological evidence for the effectiveness of chlorfenapyr. To know the efficacy of chlorfenapyr field trails are much needed.[63] |
| Gebremariam *et.al.* (2021) | Ethiopia | - DuraNet®-   **Polyethylene**  0.55% w/w ± 15% Alpha-cypermethrin | PermaNet 2.0 | Cone-bioassay | *An. arabiensis*  (Resistant strain) | DuraNet®-78% | DuraNet shows a 78% mortality rate in cone bioassays which are slightly lower than the WHO recommendations (>80%) and exhibits low to moderate efficacy rates in Ethiopia. Both nets show moderate killing efficacy[64] |
| Azizi S  *et.al.*(2021) | Tanzania | - SafeNet NF- Gram per square meter (GSM) 36 ± 10%, **Polyester,** 200 mg/m2   alphacypermethrin & Piperonyl butoxide for 100 denier.   - SafeNet®- Gram per square meter (GSM) 40 ± 10%, **Polyester, 200** mg/m^2^   alphacypermethrin & Piperonyl butoxide for 100 denier. | - Interceptor- Gram per square meter (GSM) 40 ± 10%, **Polyester,** 200 mg/m^2^ alphacypermethrin. - Untreated Safi net | Cone- bioassay | *An.*  *gambiae*  (Susceptible strain) | SafeNet NF:89.7  SafeNet:95.9  Interceptor:83.5 | SafeNet NF and SafeNet shows equivalent performance as Interceptor Net but community trials are needed to understand the durability, acceptability, and residual efficacy of SafeNet NF and SafeNet. [65] |
| Zahouli *et.al.* (2023) | Côte d’Ivoire | - PermaNet Dual - **Polyester**   5.0 g/kg ± 25% chlorfenapyr & deltamethrin 2.1 g/kg ± 25%. | - PermaNet 3.0 - PermaNet 2.0 - Untreated Net | Cone- bioassay | *An. gambiae s.l.*  (Resistant strain) | PermaNet Dual: 88.0%  PermaNet 3.0:82  PermaNet 2.0:48  Untreated Net:0 | PermaNet Dual induced high efficacy against *An. gambiae* and performed better than the PermaNet 3.0 in terms of all outcome measures. [66] |
