# Supplementary material for: Evaluation of long lasting insecticidal nets in experimental huts and WHO PQT/VCP compliance: A systematic review
Source: PLoS One. 2025 Mar 12;20(3):e0318673. doi: 10.1371/journal.pone.0318673 (PMC11902051; doi:10.1371/journal.pone.0318673)
Supplement: S2 Table — (DOCX) [file pone.0318673.s002.docx]

**Table S2. Outcome measures of Experimental hut Trials representing Deterrence, Blood Feeding Inhibition, Exophily, Personal Protection and Personal Protection reported in the included studies**

| **Ref. No.** | **Brand Name** | % | | | | | **Ref. No.** | **Brand Name** | % | | | | |
| --- | --- | --- | --- | --- | --- | --- | --- | --- | --- | --- | --- | --- | --- |
|  |  | **D** | **BFI** | **Exo.** | **PP** | **KE** |  |  | **D** | **BFI** | **Exo.** | **PP** | **KE** |
| 40 | PermaNet 3.0 | 23 | 63 | 85 | 71 | 59 | 53 | LifeNet | 21 | 96 | 59 | NIL | 70 |
|  | PermaNet 2.0 | 19 | 67 | 88 | 73 | 56 |  | CTN | 1 | 20 | 38 | NIL | 26 |
|  | CTN | 22 | 63 | 92 | 71 | 42 |  | Untreated Net | 0 | 0 | 15 | NIL | 1 |
|  | Untreated Net | 0 | 0 | 86 | 0 | 0 | 54 | MagNet | 87.5 | 48.1 | 77.8 | NIL | 13 |
| 41 | Interceptor | 2 | 65.8 | 92.6 | 75.6 | 52.2 |  | DuraNet | 87.5 | 63 | 85.2 | NIL | 12 |
|  | CTN | 3.7 | 28.3 | 92.8 | 6.4 | 57.2 |  | Untreated Net | 0 | 81.9 | 51.4 | NIL | 0 |
|  | Untreated Net | 2.8 | 0 | 86 | 0 | 0 | 55 | Dawa Plus 2.0 | 0 | 21.9 | 48.2 | NIL | 16.7 |
| 42 | Olyset Plus | Ns | 79 | 53 | NIL | 67 |  | Dawa Plus 3.0 | 0 | 29.4 | 47.9 | NIL | 17.5 |
|  | OlysetNet | Ns | 60 | 70 | NIL | 36 |  | Dawa Plus 4.0 | 10.9 | 36.9 | 50.3 | NIL | 15.4 |
|  | CTN | Ns | 74 | 65 | NIL | 55 |  | Untreated Net | 0 | 0 | 29.32 | NIL | 0 |
|  | Untreated Net | Ns | 0 | 22 | NIL | 0 | 56 | MagNet | 1.1 | 58.1 | 74.4 | 22.2 | 0 |
| 43 | OlysetNet | 78.6 | 84 | 72.6 | NIL | 62.3 |  | DuraNet | 0 | 2.6 | 78.4 | 58.3 | 4.9 |
|  | Netprotect | 83.3 | 91.9 | 55.4 | NIL | 62.7 |  | Untreated Net | 0 | 0 | 39.6 | 0 | 0 |
|  | PermaNet 2.0 | 66 | 79.8 | 66 | NIL | 62.4 | 57 | MiraNet | 63.5 | 30.6 | 57.7 | 74.7 | -2.89 |
|  | DuraNet | 81 | 87.4 | 70.2 | NIL | 74.5 |  | MagNet | 62.2 | 33.8 | 56.9 | 75 | -0.69 |
|  | Interceptor | 73.2 | 89.8 | 45.9 | NIL | 75.9 |  | Untreated Net | 0 | 0 | 26.3 | 0 | 0 |
|  | Untreated Net | 0 | 0 | 56.3 | NIL | 1.6 | 58 | Dawa Plus 2.0 | 31 | 67.7 | 58.1 | NIL | 25.8 |
| 44 | OlysetNet Duo | 0 | 75 | 56 | 71 | 48 |  | Dawa Plus 3.0 | 19 | 42.1 | 63.2 | NIL | 31.6 |
|  | Olyset Net | 0 | 15 | 53 | 3 | 27 |  | Dawa Plus 4.0 | 21 | 66.7 | 57.1 | NIL | 33.3 |
|  | Pyriproxyfen | 0 | 0 | 29 | 0 | 23 |  | Untreated Net | 26.4 | 93.9 | 23.9 | NIL | 0 |
|  | Untreated Net | 0 | 0 | 31 | 0 | 0 | 59 | Royal Guard | 9.5 | 10 | 47 | 17 | 9 |
| 45 | OlysetNet Duo | Ns | Ns | 48.6 | NIL | 14.7 |  | Dura Net | 38.7 | 7 | 47 | 43 | 4 |
|  | OlysetNet | Ns | -50.9 | 43.4 | NIL | 8.7 |  | PPF Net | 7 | 0 | 30 | 0 | 3 |
|  | Pyriproxyfen | Ns | Ns | 0 | NIL | 12.5 |  | Untreated Net | 0 | 0 | 0 | 0 | 0 |
|  | Untreated Net | Ns | 0 | 0 | NIL | 7.5 | 60 | Veeralin | 87 | 41.9 | 23 | 91.1 | 12.3 |
| 46 | ICONMaxx | 65 | 27.9 | 93.4 | 75.3 | 16.7 |  | MagNet | 84 | 39.1 | 17 | 92.3 | 8.5 |
|  | CTN | 1.8 | 0 | 91.7 | 0 | 37.4 |  | Untreated Net | 0 | 0 | 65 | 0 | 0 |
|  | Untreated Net | 0 | 0 | 90 | 0 | 0 | 61 | Yahe | 11.5 | 13.6 | 13.6 | 22.5 | 10.6 |
| 47 | Olyset Plus | 88.1 | 60.6 | 77.8 | NIL | 97.2 |  | Panda | 33.8 | 33.8 | 59.5 | 71.5 | 25.3 |
|  | OlysetNet | 82.2 | 61.9 | 70.4 | NIL | 96.3 |  | PermaNet 2.0 | -24.8 | 59.5 | 17.9 | -8.6 | 9.8 |
|  | CTN | 86.5 | 90.6 | 82.9 | NIL | 100 |  | CTN 1 | -6.1 | -3 | -3 | -10.8 | 9.4 |
|  | Untreated Net | 0 | 0 | 44.2 | NIL | 2 |  | CTN 2 | -39.7 | 6.2 | 6.2 | -33.7 | 8.4 |
| 48 | PermaNet 3.0 | 25.16 | 91.11 | 37.49 | 93.4 | 61.5 |  | Untreated Net | 0 | 0 | 0 | 0 | 8.5 |
|  | Olyset Plus | 23.44 | 85.64 | 30.49 | 89 | 55.05 | 62 | Veeralin | 2.7 | 11.3 | 75.5 | 63.1 | 8 |
|  | Yorkool | 9.89 | 61.11 | 11.27 | 65 | 40.67 |  | Permanet 3.0 | 12.3 | 13.1 | 64.3 | 61.3 | 10.4 |
|  | Untreated Net | 0 | 0 | 0 | 0 | 0 |  | Duranet | 0 | 11.8 | 76.4 | 70.3 | 0.5 |
| 49 | LifeNet | 74.8 | 63.9 | 50.7 | NIL | 91.3 |  | Untreated Net | 0 | 29.7 | 35.8 | 0 | 0 |
|  | CTN | 37.2 | 15.3 | 51.7 | NIL | 12.2 | 63 | Interceptor G2 | 4.3 | 33 | 80 | 36 | 41 |
|  | Untreated Net | 0 | 0 | 39.1 | NIL | 2.2 |  | Interceptor G1 | 30.2 | 36 | 86 | 56 | 5 |
| 50 | OlysetNet | 20.7 | 30.7 | 48.5 | NIL | 21.8 |  | CTN | 61.2 | 0 | 59 | 44 | 33 |
|  | Olyset Plus | 18.8 | 19.5 | 41.8 | NIL | 36.9 |  | Untreated Net | 0 | 0 | 42 | 0 | 0 |
|  | PermaNet 2.0 | 19.5 | 37.6 | 39.5 | NIL | 25.9 | 64 | Dura Net | No evidence | 55.94 | 36.24 | 42.4 | 54.03 |
|  | PermaNet 3.0 | 13 | 17 | 40.7 | NIL | 46.1 |  | PermaNet 2.0 | No evidence | 53.35 | 35.32 | 38.6 | 39.81 |
|  | Dawa Plus | 18.1 | 49.7 | 31.3 | NIL | 15 | 65 | SafeNet NF | 12.9 | Ns | 84.5 | NIL | 16.2 |
|  | Untreated Net | 18 | 56.4 | 26.4 | NIL | 9.5 |  | SafeNet | 3.5 | Ns | 87.2 | NIL | 15.9 |
| 51 | Dura Net | 33.25 | 96 | 89.1 | NIL | 100 |  | Interceptor | 34.1 | 35.8 | 90.2 | NIL | 24.1 |
|  | PermaNet 3.0 | 56.1 | 91.5 | 77.5 | NIL | 90 | 66 | Permanet Dual | -17.9 | 43.7 | 50.5 | NIL | 104 |
|  | Untreated Net | 0 | 0 | 30.6 | NIL | 0 |  | PermaNet 3.0 | -7.5 | 9.8 | -28.3 | NIL | 35.6 |
| 52 | Interceptor G2 | 40 | 34 | 51 | 60 | 82 |  | PermaNet 2.0 | 48 | -13 | 0.6 | NIL | 31.4 |
|  | Interceptor | 43 | Ns | 44 | 47 | 11 |  | Untreated Net | 0 | 0 | 0 | NIL | 0 |
|  | CTN | 49 | 54 | 59 | 76 | 92 |  |  |  |  |  |  |  |
|  | Untreated Net | 0 | 0 | 29 | 0 | 0 |  |  |  |  |  |  |  |

*D- Deterrence, BFI – Blood Feeding Inhibition, Exo.-Exophily, PP-Personal Protection, KE-Killing effect.
