# Supplementary material for: Evaluation of long lasting insecticidal nets in experimental huts and WHO PQT/VCP compliance: A systematic review
Source: PLoS One. 2025 Mar 12;20(3):e0318673. doi: 10.1371/journal.pone.0318673 (PMC11902051; doi:10.1371/journal.pone.0318673)
Supplement: S3 Table — (DOCX) [file pone.0318673.s003.docx]

**Table S3. WHO PQT/VCP Checklist and Number of studies adhering it.**

| **Items No.** | **Item** | **No. of Studies** |
| --- | --- | --- |
| 1 | Inclusion of study arms with candidate net, a reference net known as positive control net and an untreated net known as negative control. | 27 |
| 2 | Use of experimental huts that meet the structural specifications outlined by WHO PQT/VCP. | 27 |
| 3 | Involvement of adult human volunteers during the experimentation. | 26 |
| 4 | Employment of either the cone test or the tunnel test to assess net efficacy. | 27 |
| 5 | Reporting on the wash resistance of the candidate net. | 25 |
| 6 | Detailed explanation of the primary outcome measures used in EHTs, such as deterrence, exophily, blood-feeding inhibition, and mortality. | 22 |
