# Supplementary material for: Evaluation of long lasting insecticidal nets in experimental huts and WHO PQT/VCP compliance: A systematic review
Source: PLoS One. 2025 Mar 12;20(3):e0318673. doi: 10.1371/journal.pone.0318673 (PMC11902051; doi:10.1371/journal.pone.0318673)
Supplement: S2 File — (DOCX) [file pone.0318673.s005.docx]

**S2- FULL-TEXT ARTICLES EXCLUDED LIST, WITH REASONS**

|  | **Articles excluded** |
| --- | --- |
| Rapid/Title Screening | Tomass et.al 2016, Doda et.al 2018, Wuletaw et.al 2016, Owusu et.al 2016, Dhiman et.al 2014,Oxborough 2015 thesis, Achee et.al 2005, Picado et.l 2010, Harvey et.al 2013, Herrera-Bojórquez et.al 2020, Okumu et.al 2020, Dambach et.al 2020, Kleinschmidt et.al 2018, Janko et.al 2018, Killeen et.al 2011, Babalola et.al 2019, London et.al 2010, Phonjatturasa et.al 2021. |
| Large scale and its follow-up studies | Sharma et.al 2009,Morgan et.al 2015, Paintain et.al 2014, Omonijo et.al 2019, Sahu et.al 2020, Kilian et.al 2008, Tungu et.al 2016, Sousa et.al 2019, Raghavendra et.al. 2017, Ntuku et.al 2017, Randriamaherijaona et.al 2015, Djènontin et.al 2015, Villalta et.al 2021, Guessan et.al 2001, Mosha et.al 2022, Massue et.al 2016, Kilian et.al 2011, Gouissi et.al 2012, Malima et.al 2008, Gonahasa et.al 2018, Passah et.al 2022, Musa et.al 2020, Sagnon et.al 2015, Staedke et.al 2020,  Briet et.al 2020, Lugo Villalta et.al 2021, Toe et.al 2019, Sharma et.al 2010, Sharma et.al 2005, Corbel et.al 2010, Maiteki-Sebuguz et.al 2022, Messenger et.al 2023, Ahmadi et.al 2012, Minakawa et.al 2021, Yukich et.al 2013,Van roey et.al 2014,Martin et.al 2022. |
| Evaluation of bednets but not Phase-II evaluation | Mechan et.al 2022, Randriamaherijaona et.al 2017, Tungu et.al 2021, Fettene et.al 2009, Vinit et.al 2020, Bhatt et.al 2012, Diouf et.al 2022, Kulkarni et.al 2007, Yovogan et.al 2021, Yewhalaw et.al 2012, Okia et.al 2013, Ngufor et.al 2022, Zegers de Bey et.al 2017, Chourasiaa et.al 2017, Akoton et.al 2018, Dabiré RK et al 2006. |
| WHO reports | Guidelines for lab and field 2013, SOP for testing methods 2022, Testing procedure for Insecticide resistance 2018, Testing procedure for Insecticide resistance 2013, WHO guidelines for testing mosquito adulticides for IRS-2006, Report of WHO testing procedures 1998, WHO guidelines for efficacy testing for spatial repellents 2013, WHO Guidelines _2005, World Health Organization WHO Insecticide Resistance and Vector Control. |
| NVBDCP | National frame work for malaria, National Strategic plan, Common protocol for bio.larvicides for vector control, Compendium on entomological surveillance & Vector Control, Action plan for scaling up LLIN for malaria control 2009. |
| Other than LLINs | Darriet et.al 2000, White et.al 2018, Kitau et.al 2014, Ngufor et.al 2014, Kikankie et.al 2010, Massue et.al 2019, Malima et.al 2017, Sangoroe et.al 2014, Minakaw et.al 2007, Russell et.al 2010 |
| IRS related Studies | Ngufor et.al 2016, Rowland et.al 2013, Fongnikin et.al 2020, Oxborough et.al 2019, Yeebiyo et.al 2016. |
| Combinations of interventions | Nuwamanya et.al 2018, Ngufor et.al 2015, Djènontin et.al 2015, Ngufor et.al 2014, Ngufor et.al 2011, Cook et.al 2018, Sluydts et.al 2016, Ngufor et.al 2017, Ngufor et.al 2015. |
| Guidelines | Innovation to Impact -Dual AI ITNs, Innovation to Impact -Guidelines for Laboratory and Field-Testing of Long-Lasting Insecticidal Nets, Innovation to Impact -Net washing Guidelines, Govt. of India,MoHFW Report on Assessment of Post distribution of LLIN. |
| ITNs related studies | Asidi et.al 2005, Irish et.al 2008, Masaninga et.al 2018, Lengeler et.al 2009, Erlanger et.al 2004 |
| Comparison between LLINs and ITNs | Balodo et.al 2012, Asidi et.al 2004. |
